# Cholinergic mechanisms in spinal locomotion—potential target for rehabilitation approaches

**DOI:** 10.3389/fncir.2014.00132

**Published:** 2014-11-06

**Authors:** Larry M. Jordan, J. R. McVagh, B. R. Noga, A. M. Cabaj, H. Majczyński, Urszula Sławińska, J. Provencher, H. Leblond, Serge Rossignol

**Affiliations:** ^1^Department of Physiology and Pathophysiology, Spinal Cord Research Centre, University of ManitobaWinnipeg, MB, Canada; ^2^Department of Neurological Surgery, The Miami Project to Cure Paralysis, University of MiamiMiami, FL, USA; ^3^Department of Neurophysiology, Nencki Institute of Experimental Biology PASWarsaw, Poland; ^4^Department of Nerve-Muscle Engineering, Institute of Biocybernetics and Biomedical Engineering PASWarsaw, Poland; ^5^Groupe de Recherche sur le Système Nerveux Central and Department of Neuroscience, Faculty of Medicine, Université de MontréalMontreal, QC, Canada

**Keywords:** spinal rhythm generation, cholinergic mechanisms, *in vitro* neonatal rat, decerebrate cat, chronic spinal cat, chronic spinal rat

## Abstract

Previous experiments implicate cholinergic brainstem and spinal systems in the control of locomotion. Our results demonstrate that the endogenous cholinergic propriospinal system, acting *via* M_2_ and M_3_ muscarinic receptors, is capable of consistently producing well-coordinated locomotor activity in the *in vitro* neonatal preparation, placing it in a position to contribute to normal locomotion and to provide a basis for recovery of locomotor capability in the absence of descending pathways. Tests of these suggestions, however, reveal that the spinal cholinergic system plays little if any role in the induction of locomotion, because MLR-evoked locomotion in decerebrate cats is not prevented by cholinergic antagonists. Furthermore, it is not required for the development of stepping movements after spinal cord injury, because cholinergic agonists do not facilitate the appearance of locomotion after spinal cord injury, unlike the dramatic locomotion-promoting effects of clonidine, a noradrenergic α-2 agonist. Furthermore, cholinergic antagonists actually improve locomotor activity after spinal cord injury, suggesting that plastic changes in the spinal cholinergic system interfere with locomotion rather than facilitating it. Changes that have been observed in the cholinergic innervation of motoneurons after spinal cord injury do not decrease motoneuron excitability, as expected. Instead, the development of a “hyper-cholinergic” state after spinal cord injury appears to enhance motoneuron output and suppress locomotion. A cholinergic suppression of afferent input from the limb after spinal cord injury is also evident from our data, and this may contribute to the ability of cholinergic antagonists to improve locomotion. Not only is a role for the spinal cholinergic system in suppressing locomotion after SCI suggested by our results, but an obligatory contribution of a brainstem cholinergic relay to reticulospinal locomotor command systems is not confirmed by our experiments.

## Introduction

Acetylcholine (ACh) is thought to be a transmitter in the brainstem system for initiation of locomotion (Garcia-Rill, 1986; Jordan, 1998; Dubuc et al., 2008; Ryczko and Dubuc, 2013), and is important at the spinal level because cholinergic propriospinal cells may be involved in control of the Central Pattern Generator (CPG) for locomotion (McCance and Phillis, 1968; Huang et al., 2000; Jordan and Schmidt, 2002; Zagoraiou et al., 2009; Miles and Sillar, 2011; Tillakaratne et al., 2014). In this study we address three controversial issues: the importance of the brainstem cholinergic system in the induction of locomotion in adult animals, the capacity for the spinal cholinergic propriospinal system to provide coordinated locomotor output, and the importance of the spinal cholinergic propriospinal system in the recovery of locomotor capability in the absence of descending locomotor control.

A role for brainstem cholinergic neurons in the production of locomotion resulting from stimulation of the mesencephalic locomotor region (MLR) in a number of species is now widely accepted (Sholomenko et al., 1991; Dubuc et al., 2008; Smetana et al., 2010; Ryczko and Dubuc, 2013), but the requirement for cholinergic involvement in mammals remains controversial (McCance et al., 1968a,b; Jordan, 1998; Takakusaki et al., 2003). The MLR was originally described (Shik et al., 1966) as coextensive with the nucleus cuneiformis (CNF), but subsequent evidence has been obtained to implicate ACh, acting at muscarinic receptors, in the production of locomotor behavior in mammals (Garcia-Rill and Skinner, 1987; Garcia-Rill et al., 1987), and it has been suggested that the major output of the MLR to the reticular formation is a cholinergic projection from the pedunculopontine nucleus (PPN) (Garcia-Rill, 1986). More recent work by Takakusaki et al. (2003, 2008) compared the effects of CNF and PPN stimulation and confirmed the CNF as effective for inducing locomotion, but the PPN stimuli induces muscle tone suppression. Garcia-Rill et al. (2011), while confirming that the PPN is involved in the control of muscle tone, attempted to attribute the effectiveness of CNF stimulation for production of locomotion to the presence of cholinergic neurons within the effective sites in the CNF. If this is the case, then cholinergic antagonists should impair MLR-evoked locomotion whether the stimulus is localized to the CNF or the PPN. We elected to determine if cholinergic antagonists could alter MLR-evoked locomotion in decerebrate cats. At the same time, we tested the notion that cholinergic propriospinal neurons contribute to the normal control of locomotion in adult animals.

The mammalian spinal cord contains several types of cholinergic neurons, including motoneurons, preganglionic autonomic neurons, partition cells (lamina VII), at least two populations of central canal neurons (lamina X) and small dorsal horn cells scattered in lamina III-V (Barber et al., 1984; Houser et al., 1984; Phelps et al., 1984; Borges and Iversen, 1986). These cells are likely contributors to the effects of ACh in the spinal cord to the control of afferent input, including analgesia (Eisenach, 1999; Wess et al., 2003; Umana et al., 2013). Cholinergic cells in laminae VII and X are implicated in the control of locomotion (Huang et al., 2000; Tillakaratne et al., 2014). Cholinergic excitation of neurons involved in the control of locomotion has been demonstrated (Dai et al., 2009; Dai and Jordan, 2010), and activity of cholinergic propriospinal cells during locomotion has been documented (Webster and Jones, 1988; Carr et al., 1994, 1995; Huang et al., 2000). Activity of premotoneuron cholinergic Pitx2+ V0c neurons during locomotor episodes *in vitro* has been demonstrated (Zagoraiou et al., 2009).

There is considerable evidence that ACh can induce rhythmic activity in isolated spinal cord preparations, but usually the rhythm is not locomotor-like: although there is a left-right alternation, there is a synchronous activity in flexor and extensor motoneurons on each side. However, there are examples in the literature of well-coordinated locomotor-like activity produced by cholinesterase inhibitors, which increase release of ACh from intrinsic cholinergic neurons (Smith and Feldman, 1987; Smith et al., 1988a; Cowley and Schmidt, 1994; Kiehn et al., 1996; Anglister et al., 2008). In this study we showed that enhancing the efficacy of the propriospinal cholinergic system in neonatal animals is sufficient for production of consistent well-coordinated locomotor activity *in vitro*, and we determine the muscarinic receptors involved in this effect. These findings formed the basis for further investigation of the importance of spinal cholinergic neurons in the production of locomotion in adult animals and in animals with SCI.

Considerable attention has recently been drawn to the potential for the intraspinal cholinergic propriospinal system to influence functional recovery after loss of the descending command pathways that normally trigger locomotion. Recovery of hindlimb stepping has been related to sprouting of cholinergic fibers after SCI (Jakeman et al., 1998). It has been demonstrated that spinal motoneurons receive cholinergic C-terminals that excite motoneurons by reducing the AHP, and the cholinergic neurons that are the source of these terminals have been identified (McCance and Phillis, 1968; Zagoraiou et al., 2009; Miles and Sillar, 2011; Witts et al., 2014). Cholinergic commissural cells have also been implicated in the control of locomotion and suggested as a basis for cholinergic influences after SCI (Smith et al., 1988a,b; Martin et al., 1989; Huang et al., 2000). Changes in cholinergic propriospinal cells and inputs to motoneurons after SCI have been documented (Kapitza et al., 2012; Skup et al., 2012; Witts et al., 2014), and reduced numbers of cholinergic C-terminals onto motoneurons has been suggested as a basis for rapid exhaustion of the central drive required for the performance of locomotor movements in patients with SCI (Kapitza et al., 2012). Changes in the cholinergic control of sensory inputs would also be expected if atrophy or other alterations in cholinergic neurons involved in this process occur after SCI. Given this background of plasticity in the cholinergic propriospinal system, and its putative involvement in locomotor activity after injury, we tested whether this system is recruited during locomotor recovery in adult animals with SCI, and determined the consequences of plasticity in this system after SCI.

## Methods

Experimental procedures (neonatal rats and decerebrate cats) were approved by the University of Manitoba Animal Care Committee and conform to the standards of the Canadian Council of Animal Care. The experiments on spinal rats were carried out with the approval of the First Ethics Committee for Animal Experimentation in Poland, according to the European Union and the Polish Law on Animal Protection.

### *in vitro* neonatal rat preparations

#### Dissection

Experiments were performed on 102 Sprague-Dawley rats aged 1–4 days. Following induction of anesthesia with Halothane®, animals were quickly decerebrated and eviscerated then transferred to a 50 ml chamber lined with Sylgard® containing artificial cerebral spinal fluid (aCSF): 128 mM NaCl, 3 mM KCl, 1 μM MgSO_4_, 21 mM NaHCO_3_, 1.5 mM CaCl_2_.2HO_3_, 0.5 mM NaH_2_PO_4_.2H_2_O, and 30 mM glucose, oxygenated with 95% 0_2_/5% CO_2_. The entire spinal cord was exposed by removing the vertebral bodies, then the isolated spinal cord was placed in the bath with the hindlimbs attached (*n* = 65). In some experiments (*n* = 37) the hindlimbs were removed leaving only the isolated spinal cord, to determine whether the feedback from the attached moving limb influenced the results. In these cases, a 10 ml chamber was used. Dorsal and ventral roots were cut from cervical C1 to thoracic T12 and the surrounding tissue removed. The dorsal/ventral roots in the lumbosacral region remained intact in the hindlimb-attached preparations. When present, the pelvis was stabilized with insect pins inserted into the underlying Sylgard® to prevent movement from occurring during application of neurochemicals. Bath temperature was maintained between 5° and 19°C during surgery, while recordings were obtained at room temperature (20°–25°C). The results obtained using hindlimb—attached and isolated spinal cord preparations were indistinguishable, and the data were pooled.

#### Recordings

Electroneurograms (ENGs) were obtained using plastic suction electrodes filled with aCSF attached to lumbar roots L2 and L5 bilaterally, whether the hindlimbs were attached or not. Each ventral root recording was band pass-filtered (30 Hz to 3 kHz) and stored on a PC for subsequent analysis using software developed within our group. Details can be found at www.scrc.umanitoba.ca/doc/. Briefly, raw waveform recordings from the ventral roots were filtered at 1 Hz then rectified. Sections of the rectified waveforms containing sustained locomotor activity were analyzed in order to determine step cycle duration (onset of burst of activity in one root to the onset of the next burst in the same root) and frequency.

Circular statistics, as described previously (Kriellaars et al., 1994; Kjaerulff and Kiehn, 1996; Cowley et al., 2005; Liu and Jordan, 2005), were used to establish relationships between ventral root recordings. Briefly, we determined the relationships among locomotor-like bursts of activity recorded from pairs of ventral roots in order to compare the mean phase values of homolateral ventral roots to evaluate flexor (L2) vs. extensor (L5) coordination, as well as bilateral ventral roots, to examine right/left coordination. The angle (θ) of the mean vector represents the relationship between the reference onset and another waveform. The vector points to the direction of the maximum concentration of data points. The *r*-value, the correlation coefficient, represents the concentration of data around that vector. It is widely accepted that L2 ventral root activity consists largely of flexor motoneuron activity and L5 ventral root activity corresponds to extensor motoneuron activity (Kjaerulff and Kiehn, 1996). Cycles were selected for analysis when a sustained pattern of rhythmic activity was established.

#### Induction of rhythmic activity

Pharmacological substances were added by micropipette directly to the bath solution. The aCSF was stirred by the air mixture bubbling in the chamber. The AChE inhibitor edrophonium (EDRO), mean dose 50 μM, (25–100 μM, 10 mM stock solution) was added directly to the chamber to induce rhythmic activity. A second AChE inhibitor, neostigmine (NEO) in concentration 25 μM, was also used to confirm the effects of EDRO. Antagonists were added to the bath only when a sustained pattern of ventral root activity compatible with hindlimb stepping was produced.

#### Antagonists

Both nicotinic and muscarinic antagonists were examined in 46 preparations with well-coordinated locomotion induced by EDRO in order to determine the cholinergic receptors involved. The antagonists chosen were tubocurarine, a nicotinic antagonist (1–15 μM, *n* = 8), and the muscarinic antagonists atropine (M_1234_ antagonist, 100 nM, *n* = 5), telenzepine (M_1_ antagonist, 500 nM–20 μM, *n* = 12), methoctramine (METHOC; M_2_ antagonist, 1–50 μM, *n* = 14), 4-diphenylacetoxy-N-methylpiperidine methiodide (4-DAMP) (M_3_ antagonist, 3 nM–1 μM, *n* = 12), and muscarinic toxin-3 (MT-3) (M_4_ antagonist, 5–300 nM, *n* = 3). Statistical analysis of the effects of antagonists was carried out using Student's *t*-Test for Paired Samples. All drugs were obtained from Sigma.

### Decerebrate cat preparations

Fifteen adult cats were initially anesthetized with a mixture of nitrous oxide and Halothane® and then intubated. The left common carotid was cannulated and blood pressure monitored. A cannula was inserted into the femoral vein for the delivery of fluids and drugs. Animals were given intravenous injections of dexamethasone (4 mg, Hexadrol phosphate, Organon) to reduce brainstem swelling. The head of each animal was fixed in a stereotaxic frame with all four limbs free to step on the treadmill. The hindquarters were suspended by a sling under the belly of the cat. The animals were decerebrated at the precollicular-post-mammillary level and the anesthesia discontinued. Following a recovery period of at least 1 h, stepping on the moving treadmill was induced by electrical stimulation (square wave pulse 0.5 ms duration, 10–30 Hz, 50–200 μA) applied in the MLR (Shik et al., 1966) with monopolar steel electrodes. The electrical threshold for the initiation of hindlimb locomotion was determined by slowly increasing the strength of stimulation until locomotion ensued. Changes in the threshold for electrically induced locomotion were reported relative to the normal variability in threshold measurements observed with repeated control trials.

Locomotion of the hindlimbs was normally monitored using bilateral intramuscular electrodes for electromyographic (EMG) recordings similar to those described by English (1984) or English and Weeks (1989). Teflon-coated stainless-steel wires with exposed wire tip of 1–2 mm were implanted in various combinations of the following muscles: GL, TA, St and VL muscles. In three experiments, bilateral forelimb locomotion was also monitored with similar electrodes implanted within BB and TB muscles. The EMGs were amplified and captured using custom software.

Locomotion at four different treadmill speeds (0.3, 0.4, 0.6, 0.8 m/s) was assessed prior to and after separate intravenous doses of the muscarinic antagonist atropine (1.2–2.4 mg/kg) and/or the classical ganglionic nicotinic antagonist mecamylamine hydrochloride (3–9 mg/kg). Mecamylamine was given alone in 8 cats. The remaining 7 animals received both mecamylamine and atropine (atropine administered first in 4 animals; mecamylamine administered first in the remaining 3 animals). Mecamylamine was administered by small injections (1–3 doses of 1.0 ml) of 1.0–4.5 mg/kg/ml (injection time of <1 min). The injections of mecamylamine were given over a period of up to 50 min (Noga et al., 1987) to limit the fall in blood pressure which may be seen after its administration. Atropine was also infused gradually (over a 2–3 min period) to limit the transient drop in blood pressure which could also occur with its administration. The choice of drugs and the range of doses used was based upon the following information: (a) Renshaw cell phasic activity associated with fictive locomotion is blocked by equal or lower doses of mecamylamine (Noga et al., 1987); (b) Renshaw cell activation produced by stretch of the Achilles tendon is blocked by equal or lower doses of atropine (Ryall and Haas, 1975); (c) responses of cells recorded from a variety of locations within the brain to either iontophoretic application of acetylcholine or to electrical stimulation of afferent pathways is blocked by intravenous application of atropine at quantities similar to or less than that given in the present study (Crawford and Curtis, 1966; McCance et al., 1968b; Lake, 1973). It is clear from radiolabeled tracer studies that atropine readily passes through the blood-brain barrier (Proakis and Harris, 1978).

The following parameters were measured: step cycle length, EMG burst duration, and the number of EMG bursts per muscle per step cycle. A linear envelope signal was first obtained by passing each rectified EMG signal through an 8 or 16 Hz low pass filter. All integrated EMG channels were digitized simultaneously at 200 Hz per channel. Intralimb coordination was determined by examining the temporal relationship between EMG activity of the flexor and extensor muscles from the same limb. Interlimb coordination was qualitatively assessed (for presence of walk, trot, or gallop) and only differences following the administration of the antagonists compared to the pre-drug controls for each treadmill speed are reported. The onset for each step cycle was determined from the EMG channel with the sharpest burst onsets and most consistent activity throughout the experiment. All step cycles in each channel were then normalized, the EMG activity averaged and the standard deviations computed. The area of the EMG signal (mean and standard deviation) between the onset and offset of each burst (relative to the baseline) was calculated as a quantitative measure of muscle activity during each burst in the step cycle. The average step cycle duration and burst duration (as a percentage of the step cycle) was computed for each trial.

### Spinal cat preparations

Chronic experiments were performed in 2 spinalized cats for a period of 90 days with several injections of drugs per week. Acute experiments (*n* = 9) were performed in unanesthetized decerebrate cats a few hours after a complete spinalization (*n* = 4) or around 1 week after spinalization (*n* = 5). All procedures were conducted according to the Guide for the Care and Use of Experimental Animals (Canada), using protocols approved by the Ethics Committee of Université de Montréal.

#### Implantation of chronic EMG electrodes

All surgical procedures were performed in aseptic conditions and under general anesthesia. The cats received buprenorphine (0.01 mg/kg s.c.) 1 h before surgery. They were pre-medicated with ketamine (10 mg/kg), acepromazine maleate (AtravetTM, 0.1 mg/kg), and glycopyrrolate (0.01 mg/kg) injected intramuscularly and anesthetized by isoflurane 1–3% in 95% O_2_ maintained through an endotracheal tube. Lactate Ringer solution was administrated during the surgery through an intravenous catheter in the cephalic vein. The body temperature and heart rate were monitored during the whole surgery. The animal was secured in a stereotaxic frame. The EMG electrode implantation was as in Belanger et al. (1996) and Bouyer et al. (2001). The implanted muscles were the following: Srt, St, VL, GL, and TA. The intrathecal implantation was as in Chau et al. (1998). A fentanyl (2.5 mg, 25 μg/h) transdermal patch was fixed to the skin for 10 days after surgery. The postoperative care was as in previous publications (Belanger et al., 1996; Chau et al., 1998).

#### Recording and analysis procedures

The EMG signal was differentially amplified (bandwidth 100 Hz to 3 kHz) and digitized at 2 kHz. The EMGs were synchronized to the video images of the hindlimbs using a digital time code, recorded on the computer and on the video. Reflective markers were placed on the skin overlying the iliac crest, the femoral head, the knee joint, the lateral malleolus, the metatarsophalangeal joint and the tip of the fourth toe. Video images of the locomotor movement (left side) were captured by a digital camera and recorded on a videocassette recorder at 30 frames/s.

The bursts of activity of all EMGs were rectified and integrated to quantify their amplitude. Their onset and offset were identified automatically by homemade software and corrected manually by the experimenter in order to determine temporal parameters of each cycle. The video images were digitized and the x-y coordinates of different joint markers were obtained at the frequency of 60 fields/s by de-interlacing each video frame. These coordinates were used to calculate angular joint movements and could be displayed as continuous angular displacements or stick diagrams of one step cycle (see for instance **Figure 6**). The step length was calculated as the distance between two consecutive contacts of the same foot. The time interval between the onset and the offset of each muscle was measured and synchronized to the onset of the St EMG activity.

#### Spinalization and drug administration

Spinal cord transection was performed in aseptic conditions and under general anesthesia following a protocol established in Belanger et al. (1996) and Bouyer et al. (2001). Drug administration was made either *via* a cannula inserted at T13 with the tip at L4 (Chau et al., 1998) or in a bath over L3–L4 (Marcoux and Rossignol, 2000). For intrathecal injections, concentrations of stock solutions of drugs (atropine, 4-DAMP, dihydro-β-erythroidine, EDRO) were adjusted so that the desired total dose could be injected in a single 100 μl bolus unless otherwise indicated. The procedure was first to empty the cannula which had a dead space of 100 μl and then introduce the 100 μl of the drug and fill in the dead space again with saline.

#### Cutaneous reflex testing

In acute and 1 week spinal cats, cutaneous reflexes were tested by stimulating cutaneous nerves through cuff electrodes inserted around the Superficial Peroneal (SP) and Posterior Tibial (PT) nerves on both sides and recording EMG responses through percutaneously inserted electrodes in St, TA, GL, and Srt muscles. Reflex responses were evaluated starting 5–10 min up to 2 h after the injection of drugs. The stimulating cuffs were stable and allowed tests of threshold several times during the experiment.

### Spinal rat preparation

#### Spinal cord transection

The experiments were performed on 3-month-old WAG female rats in which, under Isofluorane anesthesia (2% and Butomidor, 0.05 mg/kg b.w.) and sterile conditions the spinal cord was completely transected at a low thoracic level (T9/10). To prevent the possibility of axonal regrowth through the cavity of the lesion, 2–3 mm of spinal cord tissue was aspirated using a glass pipette as described previously (Sławińska et al., 2012). Then the muscles and fascia overlying the paravertebral muscles were closed in layers using sterile sutures, and the skin was closed with stainless-steel surgical clips. After surgery, the animals received a non-steroidal anti-inflammatory and analgesic treatment (Tolfedine 4 mg/kg). During the following 7–10 days, the animals received antibiotics (Gentamicin 2 mg/kg and Baytril 5 mg/kg) and the bladder was emptied manually twice a day until the voiding reflex was re-established.

#### Intrathecal cannula implantation

An intrathecal cannula was implanted such that the tip was targeted to the level of L3/L4 root entrance to the spinal cord. The procedure for intrathecal cannula implantation was similar to that used previously (Majczyński et al., 2005, 2006), except in this case the cannula was inserted from the caudal end of the vertebral canal. The implantation was performed in aseptic conditions under equithesin anesthesia (i.p. 0.35 ml/100 g b.w.). A silastic cannula (0.012 in I.D. × 0.025 in O.D.) was inserted into the subarachnoid space through a small opening made at the L3 vertebral level and was pushed rostrally. The cannula was fixed by sewing it to the L4 spinous process and stabilized by suturing the overlying back muscles in place. The other end of the cannula was guided under the skin to reach the skull and connected to a custom-made adaptor cemented to the bone. After surgery, the animals received a non-steroidal anti-inflammatory and analgesic treatment and antibiotics. Two to five days after surgery the patency and correct placement of the cannula was verified by injection of 15 μl of 2% lidocaine followed by 15 μl of saline (0.9%). Drugs were injected as a bolus of 20 μl of drug diluted with sterile saline and followed by a bolus of about 15 μl of sterile saline to wash the drug from the cannula. Drug injections were separated by an interval of at least 72 h. A similar volume of sterile 0.9% saline was used as a control injection.

#### Implantation of EMG recording electrodes

Ten weeks after spinal cord transection, bipolar EMG recording electrodes were implanted in the Sol and TA muscles of both hindlimbs as previously described (Sławińska et al., 2000, 2012, 2013). The electrodes were made of Teflon-coated stainless-steel wire (0.24 mm in diameter; AS633, Cooner Wire, Chastworth). The tips of the electrodes with 1–1.5 mm of the insulation removed were pulled through a cutaneous incision at the back of animal, and each of the hook electrodes was inserted into the appropriate muscle, where it was secured by a suture. The distance between the tips of electrodes was approximately 1–2 mm. The ground electrode was placed under the skin on the back of the animal at some distance from the hindlimb muscles. The connector with the wires fixed to it and covered with dental cement and silicone was secured to the back of the animal.

#### Locomotor function investigation

The hindlimb locomotor movements of spinal rats were investigated on a treadmill where the animals were kept with their forelimbs on a platform above the treadmill and their hindlimbs were touching the moving belt. Stimulation of the tail was used to elicit locomotor-like hindlimb movements and then pharmacological agents (carbachol, and 10–15 min later atropine) were applied intrathecally (both 20 μl, 1 mM). Simultaneously with EMG recordings a digital video camera was used to record the hindlimb movements. The animals were tested before and at various times after the drug application. EMG recordings were used to determine the quality of any locomotor activity induced by the tail pinching as described before (Sławińska et al., 2012, 2014).

## Results

### *in vitro* experiments in neonatal rats

The intrinsic cholinergic propriospinal system is a potential substrate for locomotor recovery after SCI (Jordan and Schmidt, 2002), because other transmitter systems capable of inducing locomotion (e.g., noradrenergic, serotonergic, and dopaminergic) arise from brain cells forming descending pathways (Rossignol et al., 2002), leaving only intrinsic spinal circuitry to account for any recovery in cases of complete SCI such as used in the animal models described here. Thus, there is an obvious need to understand in detail the patterns of rhythmic activity which the endogenous cholinergic propriospinal system can produce, and to determine the receptor(s) involved. Further, although atropine blocks the rhythmic motor activity and excitation of motoneurons produced by cholinergic agonists, there is currently no information about the muscarinic receptor subtypes responsible for the cholinergic induction of rhythmic activity in the neonatal rat. Here we show that enhancement of the effects of the endogenous cholinergic propriospinal system with an acetylcholine esterase (AChE) inhibitor can give rise to well-coordinated locomotion, including alternation between ipsilateral flexor and extensor motoneuron groups, in contrast to the synchronous flexor/extensor activity commonly induced by application of exogenous cholinergic agonists (Cowley and Schmidt, 1994). We also provide evidence that M_2_ and M_3_ muscarinic receptors are involved in the cholinergic control of locomotor activity.

**Figure 1:**
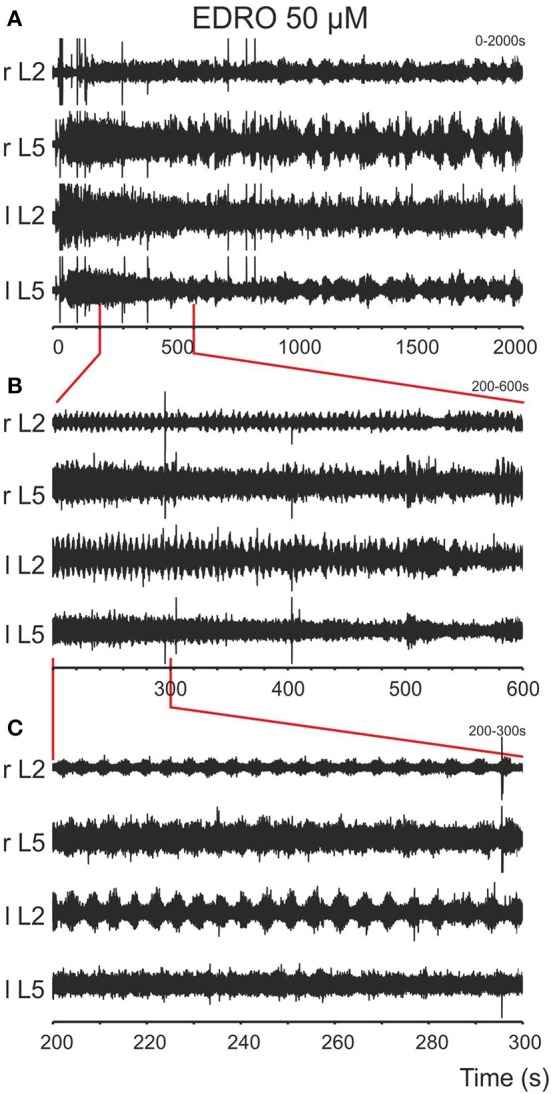
**Episodes of rhythmic activity are induced after bath application of EDRO. (A)** EDRO applied to the bath 30 s after the onset of the recording induced episodes of rhythmic activity with waxing and waning amplitude of the signal in all channels for several minutes. **(B)** Expanded sample from the same recording showing episodes of rhythmic activity (the area indicated by vertical lines) separated by periods of decreased activity. **(C)** Expanded sample of the recordings in **(B)** (the area indicated by vertical lines) showing well-coordinated locomotion characterized by alternating flexor/extensor (L2/L5) and right/left ENG activity (r/l).

**Figure 2:**
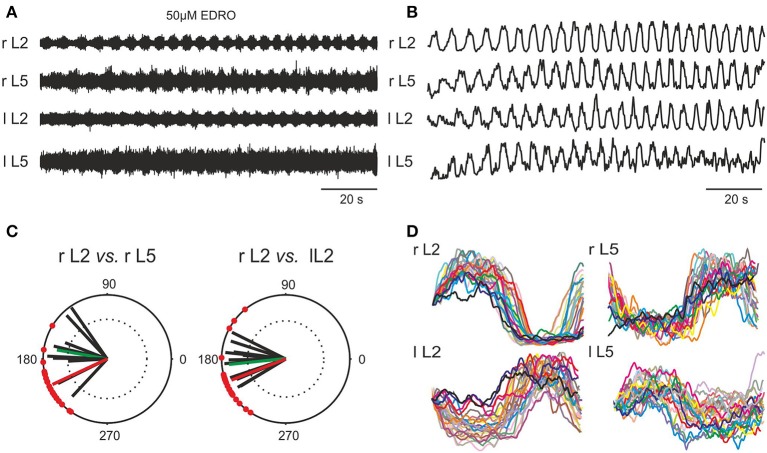
**Induction of locomotion *in vitro* by 50 μM EDRO. (A)** ENG recordings showing sustained alternating flexor/extensor and left/right activity. **(B)** Rectified and filtered waveforms of the traces in **(A)**. **(C)** Polar plots derived from pooled data from 19 experiments showing the circular distribution of mean phase values and mean angles of the right flexor ventral root ENG (r L2) vs. the right extensor ventral root ENG (r L5), and right (r L2) vs. left (l L2) flexor ENGs (green vectors). The superimposed r vectors show the concentration of phase values around the mean angle. Mean rhythm frequency (Hz) of all filtered and rectified r L2 waveforms: 0.213 ± 0.052 Hz (0.13–0.33 Hz). Mean step duration of all rectified and filtered r L2 waveforms: 4.908 ± 1.312 s (4.85–7.87 s). The phase value for ipsilateral flexor/extensor activity (θ = 205°) as well as the bilateral flexor (r L2 vs. l L2) phase value (θ = 201°) for the example shown in **(A,B,D)** is shown in **(C)** with the red vector. **(D)** Overlay of step cycles (triggered on the onset of l L2 activity) of each rectified and filtered waveform shown in **(B)**.

After baseline recordings of 30 s duration, EDRO (25–100 μM) was applied to the bath. Episodes of rhythmic activity (Figures 1A,B), with superimposed slower rhythms, were produced in 88% (64/73) of experiments after a period of 5–370 s (mean ± *SD*; 69.9 ± 75.5 s). In the remaining nine experiments, the activity was either tonic or was observed in one or two ventral roots only. In 44 of the 64 experiments in which EDRO produced rhythmic activity (69%), the activity consisted of sustained patterns of ventral root activity characterized by right/left and ipsilateral flexor/extensor alternation typical of locomotion (Figures 1C, 2). The mean number of episodes of locomotion was 6.5 per 30 min recording period. The mean duration of each episode of activity was 110.4 ± 56.1 s (range 50–184 s). The mean rhythm frequency was 0.21 ± 0.05 Hz (0.13–0.33 Hz), and the mean step duration was 4.90 ± 1.31 s (range 1.8–7.8 s).

Besides locomotion, episodes of ipsilateral flexor/extensor and right/left synchrony, episodes of ipsilateral flexor/extensor synchrony but alternating right/left sides, episodes of right/left synchrony and ipsilateral flexor/extensor alternation, or a combination of these patterns were observed, often within the same experiment. No relation was observed between the concentration of EDRO applied and the type of pattern produced in these experiments.

A second AChE inhibitor, NEO (25 μM), was also effective in inducing locomotor-like activity in the 2 experiments in which it was applied (data not shown). The mean rhythm frequency (0.22 ± 0.017 Hz), duration (4.51 ± 1.02 s) and number of episodes, 4 per 30 min recording, were similar to that produced by EDRO. However, the mean duration of each episode was 267.5 ± 488.4 s (range 20–1000 s), a more than two-fold increase over the EDRO induced locomotion. These results confirm that decreasing the breakdown of ACh in the spinal cord is sufficient to trigger locomotor activity.

In order to determine if the rhythmic activity produced by EDRO was locomotor-like, 19 of the best 44 experiments were chosen for analysis of coordination among the ventral root recordings. Raw ENG waveforms (Figure 2A) were rectified and filtered for detailed analysis to determine the relationships between flexor/extensor and right/left activity (Figure 2B). Polar plots (Figure 2C) were produced from the rectified and filtered waveforms from this example (red vectors), showing the flexor and extensor ENGs (r L2 vs. r L5) were out of phase (θ = 205°). The right and left flexor ventral root ENGs (r L2 vs. l L2) were also out of phase (θ = 201°), indicating right/left alternation. Successive steps extracted from the rectified and filtered waveforms in Figure 2B were aligned with the onset of r L2 activity and overlaid (Figure 2D) to demonstrate the consistent alternation between flexor (L2) and extensor (L5) and left (l) and right (r) waveforms. The mean rhythm frequency in this experiment was 0.21 Hz and the mean duration of each step cycle was 4.88 ± 0.64 s.

Figure 2C also shows the mean angles (green vectors) in degrees and the mean *r*-values of right flexor/extensor alternation and right/left alternation for the 19 experiments (black vectors). The overall mean angle for flexor/extensor alternation (r L2 vs. r L5) was 170.0 ± 25.4° (124.9°–206.5°), *r* = 0.84 ± 0.1 (0.66–0.98). The overall mean angle and the mean *r*-value right/left alternation (r L2 vs. l L2) was 186.1 ± 21.94° (153.6°–235.1°), *r* = 0.87 ± 0.08 (0.67–0.96). These data indicate that well-coordinated locomotion occurred consistently with EDRO enhancement of the endogenous cholinergic system, without any requirement for cross-wavelet/coherence analysis to reveal “hidden” patterns of activity, as suggested by Lev-Tov and co-workers (Anglister et al., 2008).

### The effects of ACh receptor (AChR) antagonists

#### Nicotinic antagonists

In order to determine which AChR subtype is required for EDRO induced locomotion, we conducted experiments with both nicotinic and muscarinic antagonists. The non-specific nicotinic antagonist tubocurarine was used and had no effect in 8 experiments at concentrations of 1–20 μM, up to 70 times the *K*_*i*_ of tubocurarine (280 nM) (Khan et al., 1994). It can therefore be concluded that EDRO-induced locomotion does not require nicotinic receptors.

#### Muscarinic antagonists

**Figure 3:**
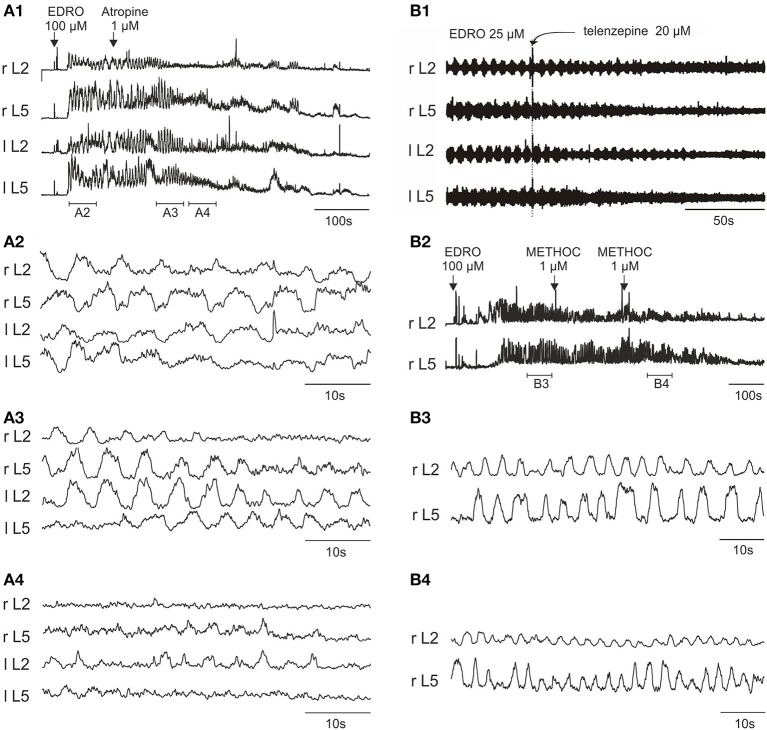
**Atropine, telenzepine, and methoctramine effects on the EDRO-induced locomotor rhythm. (A1)** Rectified and filtered waveforms showing atropine block of EDRO-induced locomotion. **(A2)** Baseline locomotor-like activity. **(A3)** Transient increase in frequency and a decrease in amplitude after 1 μM atropine. **(A4)** Progressive reduction of EDRO-induced activity by atropine. **(B1)** Telenzepine (20 μM), an M_1_ receptor antagonist, blocks EDRO-induced locomotor-like activity only at high doses, decreasing the amplitude but not the frequency of the ENG activity. **(B2–B4)** Methoctramine (METHOC, M2 receptor antagonist) produces an increase in frequency of EDRO-induced locomotion at 1–2 μM. **(B2)** Rectified and filtered waveforms recorded from the right L2 and L5 ventral roots produced by 100 μM EDRO. **(B3)** Baseline locomotor-like activity recorded during the period indicated by the horizontal bar below the ENG trace in **(B1)**. **(B4)** Increase in frequency of the ENG bursts resulting from a cumulative dose of two 1 μM METHOC (period indicated in **B2**).

To determine if muscarinic receptors have a role in EDRO-induced locomotion, we examined the effects of the non-specific muscarinic antagonist atropine. Atropine has high affinity for all M receptor subtypes. In each of six experiments atropine decreased the amplitude of ENG bursts and blocked locomotion at concentrations from 105 nM to 1 μM. This result indicates that muscarinic receptors are required for the locomotion produced by EDRO, confirming previous observations (Smith et al., 1988a). In addition, however, we found that in 4 of 6 preparations prior to blocking locomotor activity completely, atropine produced an initial increase in the frequency (not burst amplitude) of locomotion that persisted until locomotion ceased (up to 16 min after the addition of atropine). The mean frequency prior to atropine was 0.207 ± 0.06 Hz, while after atropine treatment the frequency increased to a mean of 0.28 ± 0.06 Hz. This is a highly significant change (*p* < 0.003). This effect is shown in Figure 3A. In many cases the duration of the episode was prolonged, further suggesting a facilitatory action of atropine. This was not systematically investigated, however. To determine which muscarinic receptor subtypes might account for the effects of atropine, we examined the effects of antagonists to M_1_(telenzepine), M_2_ (methoctramine), M_3_ (4-DAMP) and M_4_ (MT-3) receptors. The M_5_ receptor was not examined because M_5_ receptors have not been observed in the ventral horn of the neonatal rat spinal cord (Wilson et al., 2004).

#### M_1_ receptor antagonist

The M_1_ receptor antagonist telenzepine was used in 12 experiments, starting with concentrations of 500 nM and increasing in concentration to 20 μM. Telenzepine failed to block locomotion at concentrations of 500 nM to 5 μM, (500–5000 fold greater than *K*_*i*_). Telenzepine blocked EDRO induced locomotion only when the concentration reached 20 μM, (20,000 fold higher than *K*_*i*_). In all cases where telenzepine blocked EDRO induced locomotion, the amplitudes of the ENG bursts were decreased but the frequency of stepping was not consistently altered (Figure 3B1). This implies that the action of telenzepine was not on CPG elements responsible for producing the locomotor rhythm, but on cells involved in controlling the output of motoneurons, or on the motoneurons themselves. The high doses required for telenzepine to effectively block locomotion imply that the effect was not due to a specific action at the M_1_ receptor, but more likely because it also has affinity for the M_2_ receptor, found on cholinergic propriospinal cells and on motoneurons, or because it has affinity at higher doses for the M_3_ receptor. As noted below, the potent M_3_ receptor antagonist 4-DAMP blocks EDRO-induced locomotion at doses in the nanomolar range.

#### M_2_ receptor antagonist

We tested the M_2_ receptor antagonist methoctramine (METHOC) in 14 experiments with concentrations from 1 to 50 μM. When applied to the chamber in concentrations from 1 to 2 μM (100–200 fold higher than K_i_) METHOC seldom blocked locomotion. At these doses, METHOC produced a striking increase in the frequency of the locomotor bursts (Figures 3B2–B4). This effect was observed in 6 of 9 trials. The mean frequency prior to METHOC was 0.19 ± 0.03 Hz, while after METHOC treatment the frequency increased to a mean of 0.33 ± 0.04 Hz. This is a highly significant change (*p* < 0.001). These results suggest that METHOC relieves some form of muscarinic action that suppresses locomotion, perhaps through an inhibitory action on locomotor neurons, or through an excessive excitatory drive to CPG neurons that would serve to prolong each phase of the locomotor cycle. This is very likely through actions on the M_2_ receptors, because METHOC has a rather low affinity for M_1_, M_3_, and M_4_ receptors. These results are consistent with M_2_ receptor mediated suppression of CPG activity. At a concentration of 2–50 μM (200–3000 fold higher than K_i_) METHOC was able to suppress locomotion (5 of 9 trials). As with telenzepine, in every case where METHOC blocked EDRO-induced locomotion, ENG amplitude was decreased (Figure 3B4). This is consistent with an action directly on motoneurons, which are known to possess M_2_ receptors (Hellstrom et al., 2003). The importance of these receptors has been recently reviewed (Brownstone, 2006; Miles and Sillar, 2011; Witts et al., 2014).

#### M_3_ receptor antagonist

**Figure 4:**
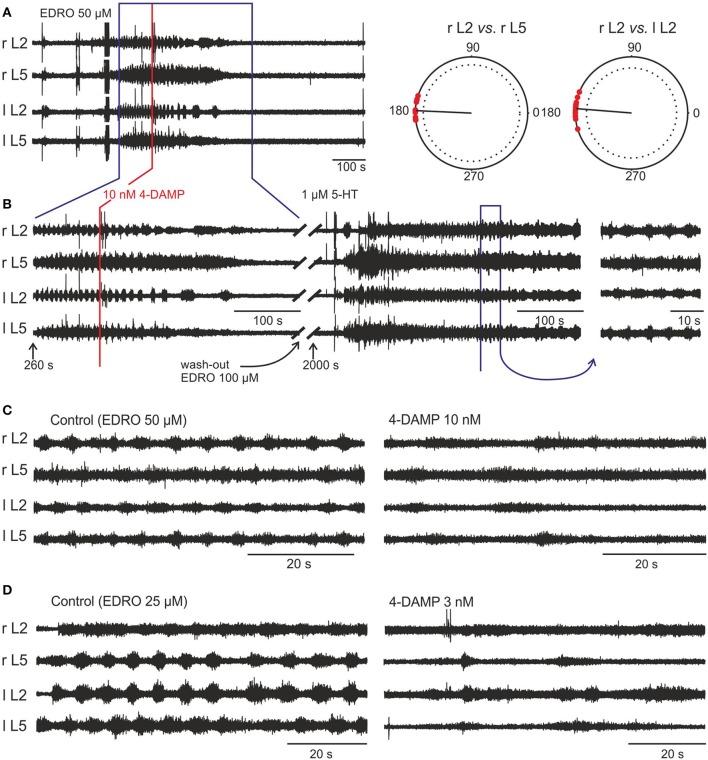
**EDRO-induced locomotion is blocked by 4-DAMP. (A)** 50 μM EDRO induced a sustained pattern of locomotion. **(A**, right panel**)** Polar Plots of flexor-extensor and left/right alternation show highly coordinated locomotion prior to the application of 4-DAMP. **(B)** 4-DAMP applied to the bath decreased rhythm frequency from 0.178 to 0.063 Hz followed by complete blockade of rhythmic activity. An additional dose of EDRO (100 μM) failed to induce locomotion (data not shown), but adding 1 μM 5-HT to the bath produced tonic firing followed by locomotor-like activity. The fact that 5-HT can “rescue” locomotor-like activity despite continued block of the EDRO effect shows that the preparation remained viable and that M_3_ receptor blockade did not interfere with locomotion induced by 5-HT. **(C,D)** Comparison of the effects of 4-DAMP on EDRO-induced locomotion with hind limbs attached **(C)** or removed **(D)**. The right panels show the results from the application of 4-DAMP in the two preparations.

The muscarinic antagonist 4-DAMP was chosen due to a reported high affinity for the M_3_ receptor subtype (Michel et al., 1989). In 12 experiments, 4-DAMP blocked locomotion at concentrations of 3, 10, 100, 500 nM, and 1 μM. Figure 4A shows a typical example of EDRO-induced locomotor activity. In this example, the hindlimbs were attached and moved during episodes of locomotor activity. Subsequent addition of 4-DAMP (10 nM) to the bath slowed the locomotor rhythm and eventually blocked it completely (Figure 4B). After wash-out, a subsequent dose of EDRO (100 μM) had no effect. In order to demonstrate that the preparation was still capable of producing locomotion, 1 μM 5-HT was then added to the bath (Figure 4B). Well-coordinated locomotion was induced, indicating that the locomotor CPG could still be activated in the absence of recovery of the EDRO effect. In each of the experiments where 4-DAMP blocked locomotion, subsequent application of EDRO, at its maximum concentration of 100 μM, failed to re-establish locomotion or induce any rhythmic activity.

In experiments where the hindlimbs were removed and 4-DAMP was applied to the isolated spinal cord alone (*n* = 8), the outcomes of 4-DAMP application on the locomotor cycle characteristics (frequency and amplitude) were similar to those obtained with hindlimbs attached (*n* = 4). Examples of 4-DAMP effects on locomotor frequency in these two preparations are shown in Figures 4C,D. The mean step frequency of EDRO-induced locomotion before 4-DAMP with hindlimbs attached (Figure 4C, left panel) was 0.176 Hz and the mean step frequency after 4-DAMP was applied and before complete cessation of locomotion (Figure 4C, right panel) was 0.068 Hz. The mean step frequency of EDRO-induced locomotion before 4-DAMP with hindlimbs removed (Figure 4D, left panel) was 0.134 Hz and the mean step frequency after 4-DAMP was applied (Figure 4D, right panel) was 0.055 Hz. ENG amplitude decreased in all experiments using 4-DAMP. This suggests that the effect of 4-DAMP may be on neurons intrinsic to the spinal cord, rather than on the relay of sensory signals from the moving limb. The decrease in step frequency is consistent with M_3_ receptor involvement in the cholinergic activation of cells within the locomotor network. Unlike atropine and METHOC, 4-DAMP did not produce even a transient increase in the frequency of locomotion.

Importantly, 4-DAMP was effective at doses as low as 3 nM. This finding demonstrates that 4-DAMP, in contrast to the M_1_ and M_2_ antagonists, blocks EDRO-induced locomotion near its *K*_*i*_, thus implicating the M_3_ receptor as the most likely muscarinic receptor subtype responsible for EDRO-induced locomotion in the *in vitro* neonatal rat preparation.

#### M_4_ receptor antagonist

MT-3 was tested in three experiments with concentrations from 5, 10, and 300 nM (1.6 fold lower and 35 fold higher than *K*_*i*_). It had no effect on EDRO-induced locomotion in any of these cases (data not shown). It is unlikely, therefore, that the M_4_ receptor is involved in the locomotor activity produced by enhancing the effects of the endogenous cholinergic propriospinal system with EDRO.

These results show that the endogenous cholinergic propriospinal system is capable of producing coordinated locomotor activity, and that under the conditions of our experiments the control of motoneuron excitability is mediated by M_2_ receptors, while the rhythm generating component is controlled by M_3_ receptors. This raises the possibility that this endogenous spinal system might contribute to the locomotion in the intact animal and to recovery of locomotion after SCI.

### Intravenous cholinergic antagonists—decerebrate cats

In order to test the importance of both the brainstem cholinergic nuclei implicated in the initiation of locomotion and the endogenous cholinergic propriospinal system we used intravenous application of cholinergic antagonists in decerebrate cats. We elicited locomotion with stimulation of the MLR. Because both nicotinic and muscarinic receptors have been implicated in the relay of the locomotor command from the MLR to reticulospinal neurons (Ryczko and Dubuc, 2013), we tested antagonists to both types of receptors. These experiments also provided a test of whether the intrinsic cholinergic propriospinal system is normally recruited as a component of the locomotor CPG in adult animals.

**Figure 5:**
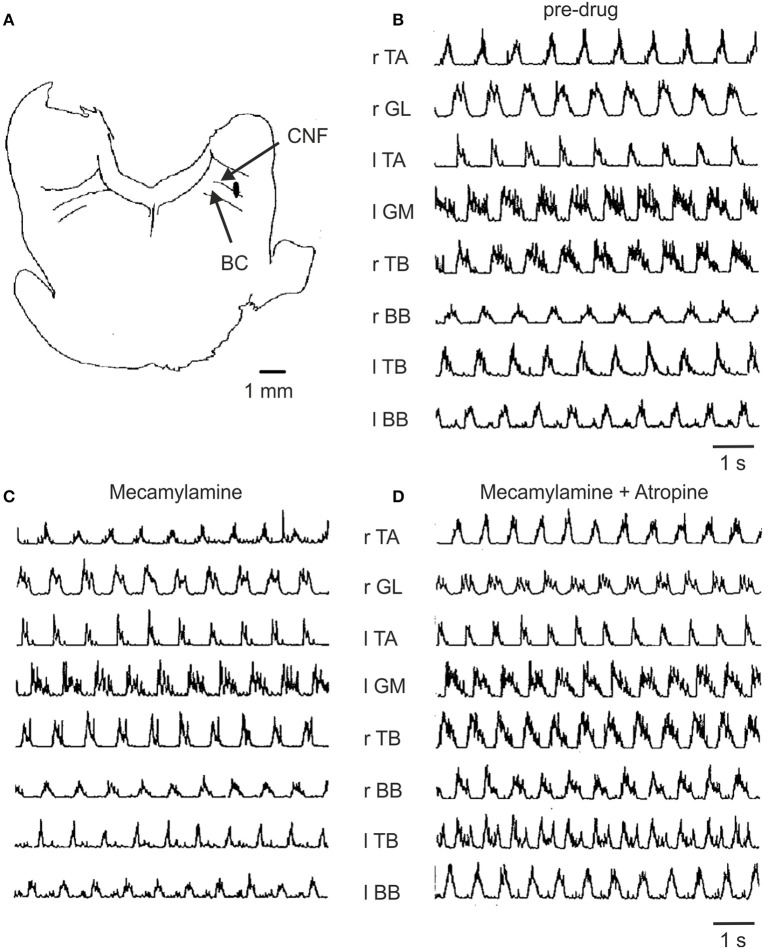
**Effect of mecamylamine and atropine on locomotion induced by electrical stimulation of the MLR. (A)** Site of stimulation was localized to the ventrolateral border of the cuneiform nucleus (CNF) near the brachium conjunctivum (BC) at P2, L4, H-1.5 (coordinates according to Berman, 1968). **(B)** Treadmill locomotion evoked from this brainstem site prior to any drugs (stimulus strength of 135 μA); **(C)** following the infusion of mecamylamine (3 mg/kg, stimulation strength 60 μA) and **(D)** following atropine (2.5 mg/kg, stimulation strength 70 μA). Thresholds for the initiation of locomotion for **(B–D)** were 150, 150, and 75 μA, respectively. The gain of each muscle's EMG is the same throughout all trials.

Previous work on decerebrate cats showed that MLR-evoked locomotion could be abolished with injections of cholinergic antagonists into a specific site in the medulla that receives cholinergic input from the MLR (Garcia-Rill and Skinner, 1987). Whether this is an obligatory route for evoking locomotion has not been tested, however. Here we show (Figure 5) that intravenous infusion of atropine and/or mecamylamine did not abolish MLR-evoked treadmill locomotion in any of the experiments (*n* = 15). Administration of mecamylamine alone produced slight increases in the threshold for electrically induced locomotion that were within the range of normal variability for lengthy experiments on decerebrate animals (114, 116, 119 and 133% of control) in 4/7 experiments. No change was observed in two experiments and a decrease in the threshold (to 63% of control) was observed in the remaining one. Atropine given alone produced a transient increase in threshold (111% of the projected baseline) in one experiment and had no effect in one other. The effect of a combination of atropine and mecamylamine on the threshold for electrically induced locomotion was examined in 5 experiments. Aside from a transient increase (5–8 min) in locomotor threshold coinciding with a drop in blood pressure, no change in threshold outside of the normal variability was observed (100, 104, 130, 131, and 131% of control trials) after administration of both drugs. In four animals brief periods of spontaneous locomotion were present both prior to and after infusion of atropine and/or mecamylamine. The threshold for electrically-induced locomotion (outside of the normal variability) was transiently increased (5–8 min) in 5 animals after administration of mecamylamine or both drugs together. Since intravenous administration of atropine or mecamylamine (at the doses used in the present study) has a prolonged effect (Ueki et al., 1961; McCance et al., 1968b; Lake, 1973; Ryall and Haas, 1975; Noga et al., 1987) these brief increases in threshold are attributed to the transient decreases in blood pressure observed following administration of these drugs. Thus, no evidence for blockage of either spontaneous or MLR-induced locomotion by mecamylamine or atropine was obtained in the present experiments.

Figure 5 illustrates the EMG activity recorded in a typical experiment prior to and after administration of mecamylamine and atropine. The lowest threshold MLR stimulation site (Figure 5A) located in the cuneiform/subcuneiform nucleus was in close proximity to the cholinergic cell group hypothesized to be functionally relevant to the production of locomotion (Garcia-Rill and Skinner, 1987; Garcia-Rill et al., 1987). The muscle activity persisted with the normal timing relationships among the muscles maintained in spite of the administration of mecamylamine (Figure 5C) and atropine (Figure 5D) at doses appropriate for the blockage of nicotinic and muscarinic transmission. In this example, the thresholds for initiation of locomotion by electrical stimulation of the MLR did not increase but rather decreased after drug administration.

In order to determine the effect of the drugs on the overall levels of motor unit activity produced during locomotion, averaged EMG records obtained following drug administration were compared to those recorded during control trials. In 285 trials where TA and GL muscle activity was examined, mecamylamine produced an increase in EMG burst activity in 3 (1.1%), a decrease in 20 (7.0%) and no change in EMG burst activity in 262 (91.9%). Of the 38 trials with atropine an increase of activity in 6 (15.8%), a decrease in 3 (7.9%) and had no effect in 29 (76.3%) was observed. Combined administration of atropine and mecamylamine produced an increase in muscle activity in 14/142 trials (9.9%), a decrease in 5/142 trials (3.5%) and had no effect on muscle activity in 123/142 trials (86.6%). It is clear from these data that the amount of muscle activity as measured from the area of the burst during the normalized step cycle was not altered in any consistent manner by cholinergic blockade.

We examined the effects of the drugs on the burst duration and times of onset and termination of EMG activity during the step cycle. In 315 trials on 6 muscles (TA, GL, IP, AF, TB, and BB), mecamylamine produced an increase in burst duration in 81 (25.7%), a decrease in 60 (19.1%) and no change in 174 (55.2%). For 88 trials, atropine produced an increase in burst duration in 33 (37.5%), a decrease in 11 (12.5%), and had no effect on burst duration in 44 (50.0%). It is evident from this analysis that cholinergic blockage did not consistently alter the duration of EMG activity in any of the muscles examined.

These data reveal that in adult decerebrate cats there appears to be no requirement for cholinergic neuron participation in the initiation or coordination of locomotion at either the brainstem or the spinal cord levels, using i.v. application of drugs known to cross the blood-brain-barrier for testing the actions of muscarinic and nicotinic receptors. In agreement with this, it appears that the cholinergic C-boutons on motoneurons do not provide a major component of the locomotor drive (Witts et al., 2014), consistent with our finding that blockage of muscarinic inputs to motoneurons in adult cats does not alter the amplitude of EMGs recorded from hindlimb motoneurons. It is still possible, however, that cholinergic neurons of the spinal cord participate in the recovery of locomotor activity after SCI. Since this propriospinal system is a major intrinsic system known to facilitate locomotion that is spared by SCI, we hypothesized that it could be responsible for locomotor recovery. To establish this, we examined the effects of intrathecal cholinergic agonists and antagonists on locomotor recovery in cats and rats after spinal cord transection.

### Spinal cats

An initial set of experiments was conducted in 2 spinal cats prepared so that intrathecal (i.t.) injections of various agonists (carbachol) or the AChE inhibitor EDRO, as well as antagonists such as atropine, 4-DAMP and dihydro-β-erythroidine could be injected in volumes of 100 μl (unless specified otherwise) in both cats on alternate days, thus keeping the time sequence of injections after spinalization approximately the same.

#### Early chronic period (5–14 days)

**Figure 6:**
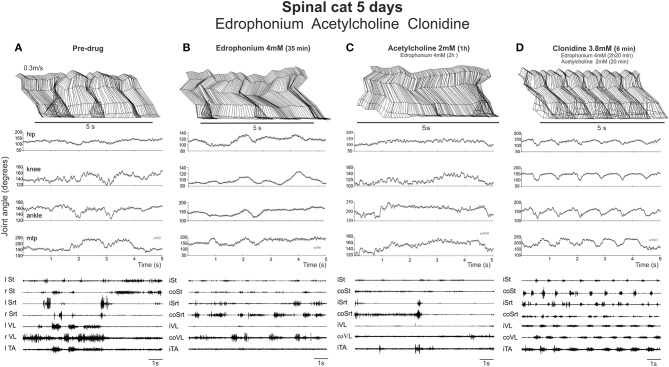
**Neither EDRO nor ACh in the presence of EDRO facilitated the onset of locomotor activity in a spinal cat. (A)** Pre-drug records of a cat walking on a treadmill with strong exteroceptive stimulation used to activate the locomotor CPG. Stick figures of the limb trajectory over 5 s of activity, along with the corresponding joint angles and EMG recordings are illustrated. **(B)** Similar records taken 35 min. after 4 mM EDRO administration. **(C)** Recordings taken 1 h after administration of 2 mM ACh and 2 h after the original dose of edrophonium. **(D)** Subsequent dose of clonidine (3.8 mM) elicited typical stepping movements on the treadmill, as illustrated by joint angles and EMG, showing that the animal was capable of producing locomotion.

On Day 5 and 6 post lesion, the animal was placed on a treadmill running at 0.3 m/s. As expected, only faint movements were evoked by strong perineal stimulation and sporadic tonic hyperflexion of both hindlimbs but without any locomotion. This is illustrated in Figure 6A where stick figure reconstructions of the movements over consecutive seconds is shown (top panel), as well as the corresponding angular excursion of the joints (middle panel) and EMGs (bottom panel).

EDRO was then given i.t. (each application in a bolus of 100 μl) in two cats **(**in one 0.5 mM then another 0.5 mM 13 min later, in another 1 dose of 2 mM directly) and the effect monitored each 10 min over a 30–40 min period by holding the cat over the treadmill belt and by applying various skin or perineal stimulation to facilitate locomotor movements. No obvious effect was observed after EDRO (compare Figures 6A,B). Acetylcholine was then given (in one cat 1 mM; in the other 2 mM) 45 min after EDRO and again, no locomotor movements were observed (Figure 6C) for the next 1:30 h. To test the ability of the spinal cat to generate locomotion, 100 μg of clonidine was given i.t. and vigorous stepping was triggered for the next 2 h, from 0.3 to 0.7 m/s. As shown in Figure 6D, 6 min after clonidine, alternation between extensor and flexor muscles in each limb and alternation of the activity of right and left homologous muscles lead to a regular pattern of locomotion on the treadmill involving all 4 joints. The cat walked with the limb somewhat in extension as should be expected only 5 days after spinalization. This was seen in both cats and was repeated the day after (6 days post lesion) in one cat with a higher dose of EDRO (4 mM) and higher doses of acetylcholine (4 mM). In the other cat, higher dose of EDRO (4 mM) only, without acetylcholine, was tested to observe the effect of the acetylcholinesterase alone and again, no obvious effect was observed while clonidine could still trigger locomotion when injected after all the other drugs. It is thus clear that the AChE inhibitor produces no significant effect on the initiation of locomotion in spinal cats, unlike its effects in the isolated neonatal rat spinal cord. ACh deteriorates the already poor stepping and clonidine evokes a sustained locomotor pattern indicating that cats were capable of walking and suggesting that the negative effects obtained with cholinergic stimulation was not due to the inability of the spinal cats to walk.

**Figure 7:**
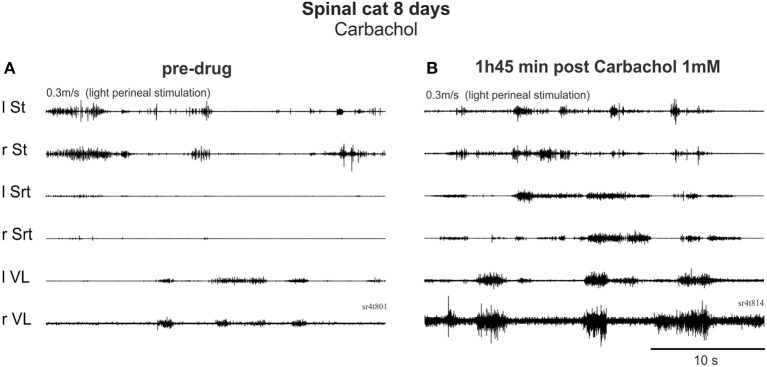
**Carbachol, a cholinergic agonist, produced co-contractions and interfered with locomotor activity early after injury. (A)** Pre-drug trial with EMG activity produced by perineal stimulation. **(B)** 1 h 45 after i.t. carbachol (1 mM, 100 μl) administration, perineal stimulation produced uncoordinated rhythmic activity.

*On Day 8*, after only 1 day of training (3 times for 5 min), the cholinergic agonist carbachol (1 mM) was tested in both cats. Before drug administration, one cat could make a few steps on the treadmill with foot contact on the dorsum of the foot at 0.3 m/s (not shown). After carbachol, standing was exaggerated with hyper-extensions of the joints and stepping was completely blocked within 7 min. Thirty minutes later, perineal stimulation evoked hyperflexions and a few steps with prolonged extension but this type of movement was far from the locomotor pattern observed before drug. In the other cat, locomotion was still not possible before carbachol (Figure 7A). With carbachol, perineal stimulation produced long duration discharges (several seconds) of several muscles often simultaneously (Figure 7B). Overall, carbachol administration was tested 4 times in the two cats and it is clear that carbachol could not trigger locomotion and was even detrimental to the faint spontaneous locomotor pattern in the cat when present.

**Figure 8:**
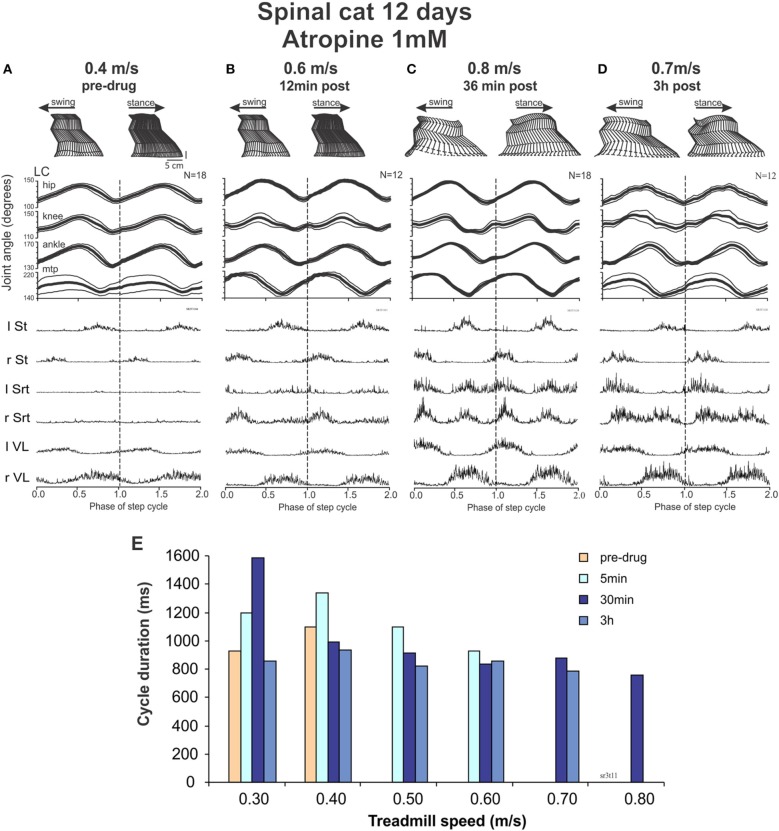
**Intrathecal atropine facilitated locomotion in spinal cats. (A–D)** show kinematic and EMG recordings of a cat walking on a treadmill at increasing time after atropine (1 mM, 100 μl) administration: stick figures of one step cycle (top), averaged angular excursion of the four joints over *N* > 12 step cycles (middle) and corresponding averaged rectified EMG traces (bottom). The cycle is normalized to 1 and is repeated twice (separated by doted vertical lines) for clarity at turning points. The average is synchronized with the contact of the left foot (LC). **(A)** Pre-drug recordings at a treadmill speed of 0.4 m/s; **(B)** 12 min after atropine administration at a treadmill speed of 0.6 m/s; **(C)** 36 min after atropine at a treadmill speed of 0.8 m/s; **(D)** 3 h after Atropine at a treadmill speed of 0.7 m/s. **(E)** Cycle duration as a function of treadmill speed, showing a maximal increase in treadmill speed the cat could follow after atropine at approximately 30 min, with a slight decrease at 3 h after drug administration.

*On days 12–13*, the cats began to show some recovery of locomotion (a few steps at 0.3–0.4 m/s) when the perineum was stimulated. At a higher speed, the animal showed some hyperflexion of both hindlimbs even with stronger perineal stimulation. As illustrated in Figure 8A, at a treadmill speed of 0.4 m/s, the cat cannot place its paw in front of the hip at the end of the swing phases and there is a paw drag in the first part of the swing phase (Figure 8A). The averaged EMGs show that although there is a good alternation between flexor and extensor muscles, like St and VL, Srt muscles on both sides have a very weak level of activity.

With this background of spontaneous activity, atropine was injected with the hypothesis that the deterioration of walking seen with ACh, carbachol or EDRO early after spinalization might be due to an already extant hyper-cholinergic state. Twelve minutes after drug administration (Figure 8B), the cat not only showed a better pattern of locomotion, it could express locomotion without the need of perineal stimulation. It could walk up to speed of 0.6 m/s. After 36 min the cat could reach treadmill speeds up to 0.8 m/s (Figure 8C). These effects could still be seen after 3 hours (Figure 8D). Figure 8E illustrates the speeds that could be reached by the cat at various time points after atropine injections. The averaged EMGs show a clear increase in the Srt muscles resulting in a greater flexion of the knee and a better elevation of the foot in the second portion of the swing phases before the paw contact, as illustrated. When carbachol was added, the spontaneous stepping was degraded although a few steps could be triggered with strong perineal stimulation at a max speed of 0.5 m/s without adequate limb extension or placement of the paws (data not shown).

Thus, at 12 days the cat is already capable of performing a few spontaneous steps. With atropine locomotion became more vigorous and 36 min later the cat could walk up to 0.8 m/s. Carbachol dramatically degraded this locomotor activity.

#### Day 14: carbachol followed by atropine

**Figure 9:**
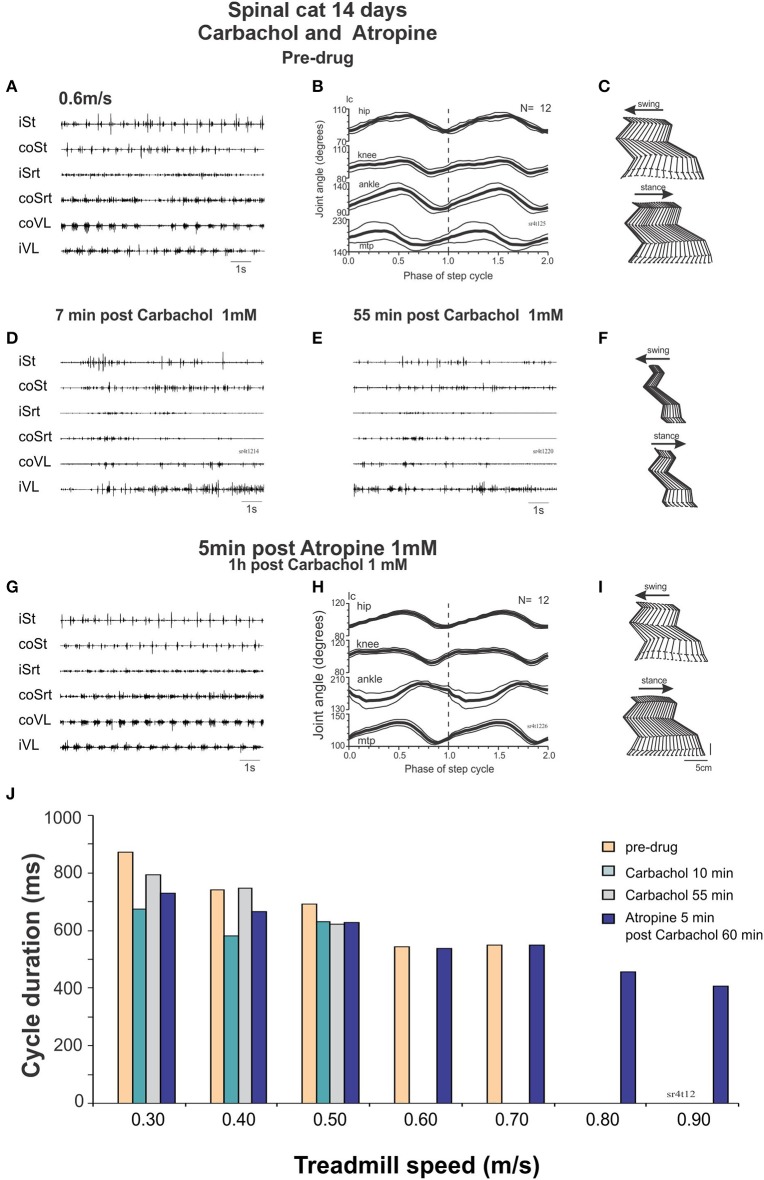
**Carbachol disruption of locomotion in spinal cats is reversed by atropine. (A–C)** regular stepping movements before drug administration 14 days after the lesion represented by raw EMG traces **(A)**, averaged (*N* = 12 cycles) joint angular displacements **(B)** and stick figures **(C)**. **(D)** Raw EMG recordings after intrathecal carbachol (1 mM, 100 μl) showing deterioration of stepping as soon as 7 min post drug administration even with strong perineal stimulation. **(E,F)** 55 min post carbachol, the EMG is still not organized to produce a good stepping pattern **(E)**. There is no foot placement as represented by the stick figures **(F)**. **(G–I)** Five minutes after intrathecal administration of atropine (1 mM, 100 μl), a good pattern of stepping was observed without perineal stimulation as was the case before carbachol administration [compare **(G–I)** with **(A–C)** same display]. **(J)** Cycle duration expressed in terms of treadmill speed, showing disruption of locomotion by carbachol, and facilitation of locomotion with atropine.

At Day 14, the spinal cats could walk spontaneously (without drugs) up to a treadmill speed of 0.7 m/s. After carbachol (Figure 9) the steps deteriorated with less weight support and no foot placement at 0.3 m/s and perineal stimulation was required to improve stepping at speeds ranging from 0.3 to 0.7 m/s. Twenty minutes after carbachol the cats could walk from 0.3 to 0.7 m/s with perineal stimulation but could not walk without it at 0.4 m/s.

This deterioration of locomotion by carbachol could be reversed by atropine. The cat could indeed walk from 0.4 to 0.6 without perineal stimulation with more forward movements of the hindlimbs. Twenty minutes later the cat could walk as fast as 0.9 m/s without perineal stimulation (Figure 9J). Thus, in a walking spinal cat, carbachol disrupts locomotion but the locomotor ability could be rescued by injecting atropine.

#### Late chronic period (>2 months)

In the late period (around 2 months) we injected a muscarinic blocker, 4-DAMP or a nicotinic antagonist, dihydro-β-erythroidine. At this time, the cat still had appreciable locomotor activity in both hindlimbs, however, they needed more perineal stimulation. After injecting 4-DAMP (45 μg in 100 μl, M_3_ muscarinic receptor antagonist), the locomotor activity was much improved, several sequences without perineal stimulation could be obtained, and the cat could often walk without any external weight support. Similar results were obtained using dihydro-β-erythroidine (26 μg in 100 μl i.t.), which produced long sequences of unaided spinal stepping. This was observed in 5 trials in 2 chronic spinal cats (40–97 days post SCI). Thus, the cholinergic suppression of spinal locomotion involves both muscarinic and nicotinic receptors (data not shown).

### Chronic spinal rats

**Figure 10:**
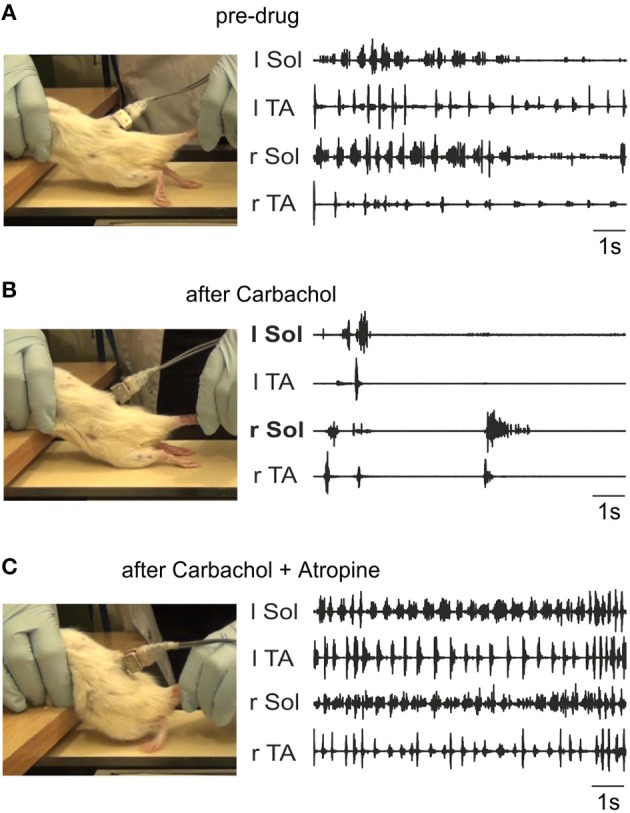
**Carbachol disruption of locomotion in spinal rats is reversed by atropine**. The left panels show representative frames from the videos taken during the time of the corresponding EMG activity (right panels). **(A)** Irregular stepping movements produced with tail stimulation prior to drug administration. As shown in the left panel, the EMG activity did not give rise to plantar stepping. The toes were always dragging on the treadmill surface during rhythmic alternating activity. **(B)** Intrathecal carbachol (1 mM, 20 μl) eliminated all stepping movements immediately, and only a few sporadic episodes of largely synchronous bursting occurred (recording taken 1 min after administration). **(C)** The intrathecal administration of atropine (1 mM, 20 μl) 10 min after carbachol treatment restored alternating movement and led to occasional plantar stepping (left panel). The EMG activity was more consistent, and occurred even in the absence of tail stimulation. The recording was made 30 min after atropine was given.

The above results indicate that despite the ability of endogenous release of ACh in the neonatal rat cord to give rise to well-coordinated locomotion, our hypothesis that locomotor recovery after SCI could be mediated by increased activity of the intrinsic cholinergic propriospinal system was not confirmed. In fact, the data suggest that a hyper-cholinergic state might develop after SCI that somehow interferes with locomotion. In order to test whether this is a consistent feature of SCI across species, and to rule out the possibility that our results in adult spinal cats might deviate from our expectations based on the neonatal rat data due to a species difference, we investigated the effects of intrathecal applications of cholinergic drugs in adult spinal rats 10 weeks after injury. Figure 10 shows a typical example of the effects of i.t. carbachol (1 mM, 20 μl) followed by an application of atropine (1 mM, 20 μl). We consistently observed a dramatic and immediate cessation of treadmill locomotion induced by tail pinching when carbachol was administered (*n* = 3) that lasted for several hours. A subsequent i.t. application of atropine (in 10–15 min after the carbachol) reversed this effect and actually increased the efficacy of tail stimulation to elicit locomotion. Plantar stepping was occasionally induced (Figure 10C), and rhythmic activity persisted even when there was no tail stimulation. Doses of 20 μl of carbachol of 0.5 and 0.25 mM were also effective. Thus, the effects of carbachol and atropine in the spinal rat were remarkably similar to the effects of these drugs in the adult spinal cat (Figure 9).

Therefore, our data provide evidence for a hyper-cholinergic state in both cats and rats after SCI, with no evidence for a species difference that might account for the unexpected absence of the locomotion promoting action of the cholinergic propriospinal system observed in neonatal rat preparations. An obvious difference between these two preparations is the presence of cutaneous receptor feedback (Bouyer and Rossignol, 2003a,b; Sławińska et al., 2012) in the spinal rats and cats walking on a treadmill. The proprioceptive feedback from the moving limb (Pearson, 2004) in neonatal rat preparations with the hindlimbs attached is not sufficient to account for the differences, because the results were not distinguishable from preparations with no hindlimbs attached. The other difference between these two preparations might be that some plasticity in the cholinergic system and the changes in the receptors induced by total transection in spinal chronic rats are not present in neonatal spinal cord preparations.

### Acute spinal cats

Our experiments on chronic spinal animals led us to attempt to determine whether the atropine effect could be observed acutely after spinalization, or whether a period of time was necessary for it to develop. We also wished to examine the actions of cholinergic drugs on sensory input, since our results suggest that a decreased response to sensory inputs may be one explanation for the detrimental effects of cholinergic agonists and the facilitation of locomotion produced by cholinergic antagonists.

#### Effect of atropine alone

In all cats, atropine alone, whether given intrathecally (1 mM, 70 μl) at mid lumbar segments or in a bath covering L3-L4 spinal segments, failed to evoke locomotion in acutely spinalized cats or in 1-week spinal cats decerebrated on the day of the experiment. This suggests that some plasticity in the cholinergic system is required for the effects of cholinergic drugs in chronic spinal animals (data not shown).

#### Effect of atropine on sensory input

**Figure 11:**
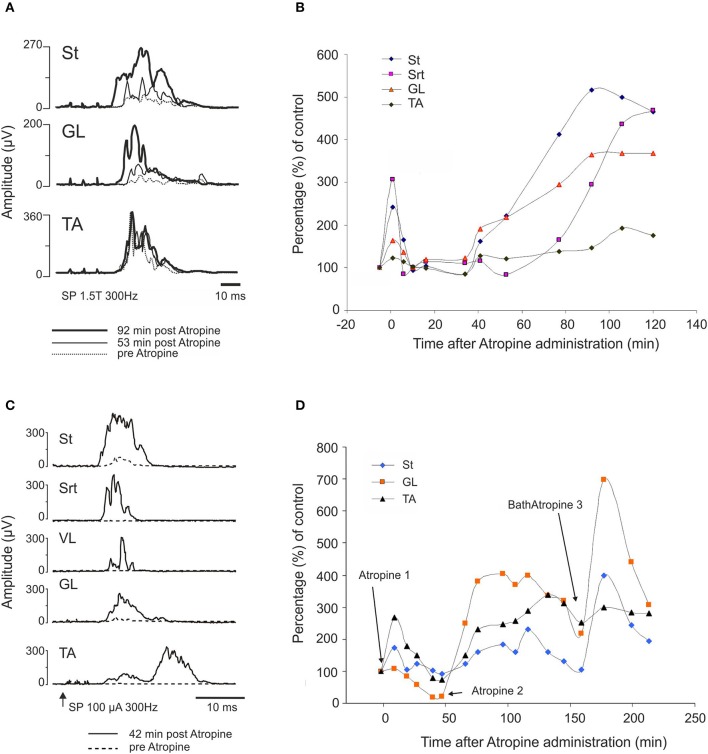
**Atropine facilitates the cutaneous reflex responses evoked by stimulation of superficial peroneal (SP) nerve. (A,B)** SP facilitation by intrathecal atropine (70 μg, 100 μl) 1 day after the complete section of the spinal cord: **(A)** averaged (*n* = 25) rectified response in ST, GL, and TA muscles after stimulation of SP (1.5T, 3 pulses, 300 Hz) 300 Hz before (dotted line), 53 (thin gray line) and 92 (thick black line) min post atropine; **(B)** illustrates the evolution of the response amplitude of the SP reflexes as a function of control responses in 4 muscles on the side of the stimulation. **(C,D)** SP facilitation by atropine 1 week after SCI. Atropine 1 and 2 correspond to intrathecal atropine (70 μg, 100 μl) whereas Bath Atropine 3 corresponds to a bath application of 1.5 ml of a solution containing 1 mg/ml of atropine.

The effect of atropine on the SP reflex was tested in 4 cats: acute (*n* = 2), 24 h (*n* = 1) and 7 days (*n* = 1) after complete section of the spinal cord at T13. The amplitude of the reflex evoked by a stimulation of the SP nerve increased after atropine (i.t., 70 μg in 100 μl) compared to control values. Figure 11A illustrates the averaged (*n* = 25) rectified response in St, GL, and TA muscles after stimulation of SP 1.5T, 3 pulses, 300 Hz before (dotted line), 53 (thin gray line) and 92 (thick black line) min post atropine in a cat spinalized 24 h before. Not only did the reflex increase in amplitude, but the latency decreased in St and GL.

Figure 11B illustrates the evolution of the response amplitude of cutaneous reflexes as a function of control responses in 4 muscles on the side of the stimulation. Amplitude of St and Srt responses significantly increased 200–300% above control level early after injection of atropine. There was clearly also a second phase of increased amplitude starting at about 40 min and lasting for at least 2 h after atropine injection.

Similar results were obtained in a 1 week spinal cat in which atropine was applied over the L3-L4 segments. In this case, the increase in reflex amplitude was obtained earlier, i.e., 42 min after intrathecal administration of atropine (Figure 11C). In the other 1 week spinal cat, the increase was obtained within the same delay as in the 24 h spinal cat, i.e., around 90 min (Figure 11D). We tested whether locomotion could be evoked by administration of a second dose of atropine in the lumbar bath. As illustrated in Figure 11D, each time atropine was given, the reflex amplitude increased (see at 180 min). However, as was the case in all cats detailed above, even this larger dose of atropine and with strong exteroceptive stimulation, no locomotion could be evoked. To test again whether this spinal cat was able to walk at all, clonidine (150 μg/kg, i.v.) was given and within a short time the usual nice locomotor pattern was evoked and reflex threshold increased from 170 to 500 μA as seen before with clonidine (Barbeau et al., 1987).

#### Atropine facilitates clonidine induction of locomotion

The previous results suggest that perhaps atropine and clonidine could have a synergistic effect, even at times after transection when atropine alone is ineffective. In one cat bath application of atropine was followed, 40 min later, by a small dose of i.v. clonidine. As was the case with the 4 above mentioned cats, atropine at L3-L4 did not evoke locomotion but, a small dose of clonidine (60 μg/kg, i.v.) 40 min after the atropine triggered a vigorous pattern of locomotion. It thus seems that even if atropine alone does not evoke locomotion, it acts synergistically with clonidine since a minimal dose of clonidine that normally fails to evoke locomotion can trigger the locomotor rhythm.

#### Effect of clonidine followed by atropine

**Figure 12:**
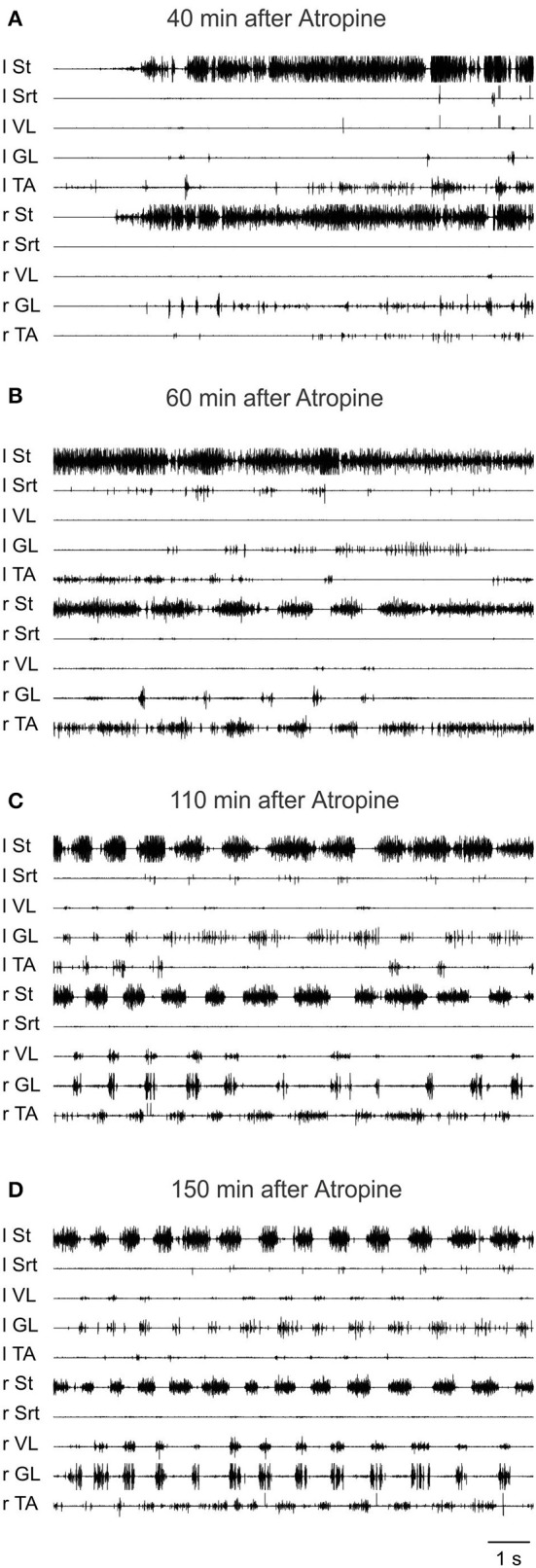
**The effect of a sub-threshold dose of clonidine is facilitated by atropine**. Clonidine was first given i.v. (20 μg/kg) and then atropine (1.5 ml of a solution containing 1 mg/ml of atropine) was given through a bath covering the L3-L4 segments of the spinal cord 40 min later in a 4 days spinal cat. **(A)** EMG signals from hindlimbs muscles 80 min after clonidine injection and 40 min after atropine application. Some bursts of activity appear after atropine especially in the hip flexors (St); **(B)** 60 min after atropine some alternating activity appears between flexor and extensor muscles but it was not organized enough to trigger strong locomotion; **(C,D)** strong pattern of locomotion was observed 110 and 150 min post atropine.

**Figure 13:**
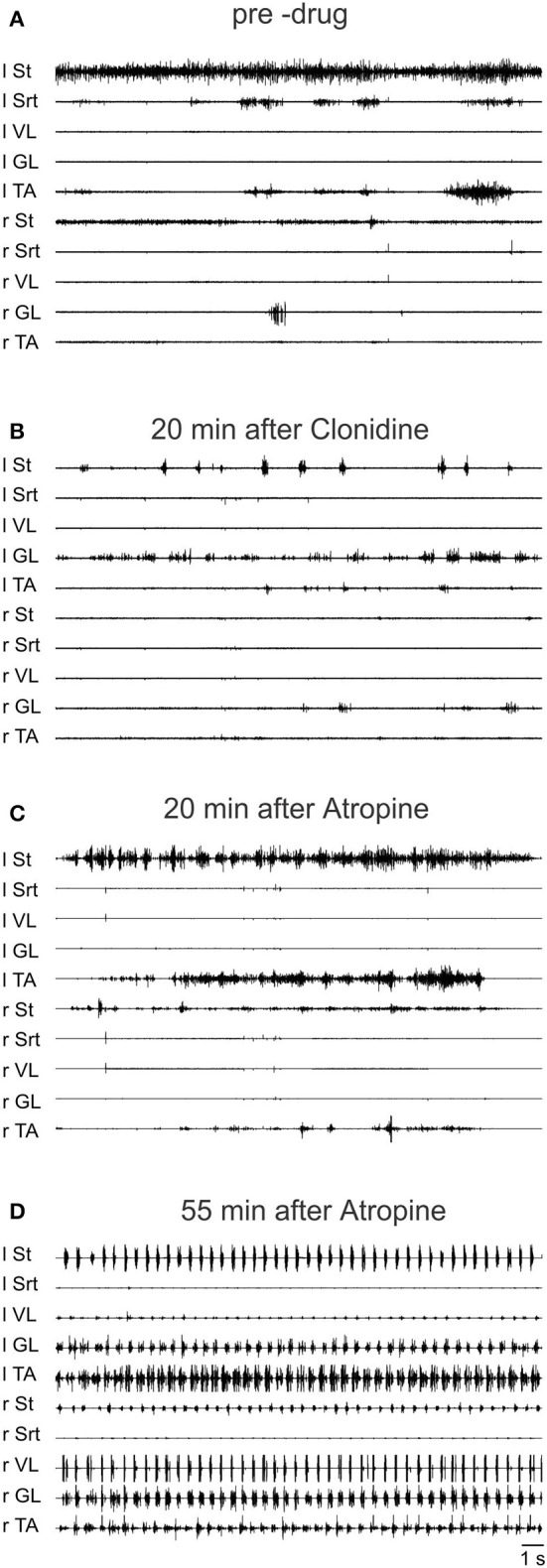
**Sub-threshold clonidine is facilitated by atropine in an acute spinal cat. (A)** Pre-drug records of EMG signals from hindlimbs muscles; **(B)** 20 min after administration of a small volume of clonidine i.v. (20 μg/kg); **(C)** 20 min after bath atropine (1.5 ml of a solution containing 1 mg/ml of atropine) over L3-L4 and 40 min post clonidine; **(D)** 55 min after atropine application, a vigorous pattern of locomotion is represented by the EMG signals.

To further validate the facilitation of locomotion produced by atropine, an even smaller sub-threshold dose of clonidine was first given (20 μg/kg, i.v.) and then atropine was given through a bath over L3-L4 in a 4 day spinal cat, a 5 day spinal cat as well as in an acute spinal cat. In all cases the dose of clonidine was insufficient to evoke locomotor rhythm by itself. Figure 12 shows the EMG activity 80 min after clonidine injection and 40 min after atropine application. At 40 and 60 min post atropine some bursts of activity appear, but strong locomotor activity was observed only 110 min post atropine (160 min post clonidine). Similar results were found in another semi-chronic cat and in one acute spinal cat (Figure 13). This late activation of locomotion corresponds to the time of the peak reflex amplitude observed in other cats with atropine.

A plausible explanation for these results is that early after SCI atropine is ineffective alone due to the absence of some plasticity that increases muscarinic control of cutaneous input after chronic injury. In the presence of low-dose clonidine, however, we propose that a tonic cholinergic control of afferent input that suppresses feedback necessary for locomotion induction is already present. Atropine blocks this suppression and thus facilitates locomotion.

## Discussion

**Figure 14:**
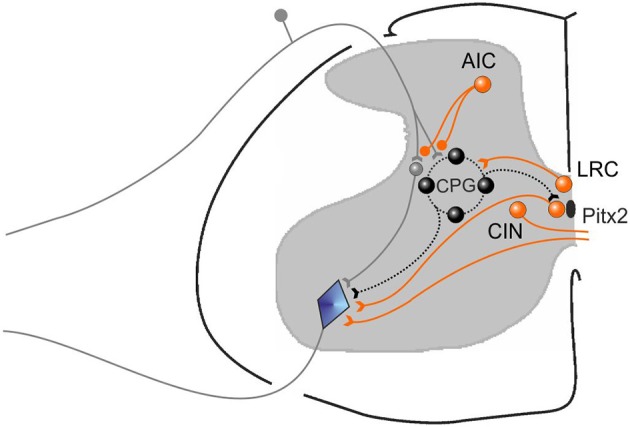
**Cartoon showing different functional groups of cholinergic neurons that are suggested by our results**. A population for afferent input control (AIC) is illustrated, some of which are known to have direct presynaptic terminals on cutaneous afferents, as well as terminals on GABAergic interneruons (not shown) that may also be responsible for suppressing afferent input. AIC neurons act on their targets through both muscarinic and nicotinic receptors. A population of cholinergic neurons (LRC) that can control the locomotor rhythm by activating the central pattern generator for locomotion (CPG) are also shown. Their precise positions are unknown. They act on their target neurons via M3 receptors. Pitx2 neurons give rise to C terminals on motoneurons and increase motoneruron excitability by reducing the AHP amplitude via M2 receptors. They are also known as V0c neurons. Commissural cholinergic interneurons (CIN) are illustrated, many of which terminate on contralateral motoneurons and produce EPSPs in motoneurons via M-currents blocked by atropine. Other partition or central canal cluster cells with unknown function are likely also present in the spinal cord.

We first demonstrated in neonatal rat preparations that facilitation of the endogenous cholinergic propriospinal system can result in coordinated locomotor activity, suggesting it may be a substrate for recovery of locomotion after SCI. We demonstrated that specific muscarinic but not nicotinic receptors are involved in this process. We tested the importance of brainstem and spinal cholinergic systems in the initiation of locomotion from the MLR in adult decerebrate cats, and showed that systemic application of neither nicotinic nor muscarinic antagonists could interfere with MLR-evoked locomotion. We tested the hypothesis that cholinergic propriospinal cells contribute to the recovery of locomotor activity in spinal cats, and found that this hypothesis was not supported. Surprisingly, both nicotinic and muscarinic cholinergic antagonists facilitated locomotion, suggesting the development of a hyper-cholinergic state after SCI. The effects of carbachol and atropine were confirmed in chronic spinal rats, showing that the results obtained in the adult spinal cat were not due to a species difference. Finally, we provided evidence that an underlying change in cholinergic contributions to spinal control of locomotion was facilitation of the control of afferent input, so that cholinergic antagonists were effective facilitators of spinal locomotion. Different populations of cholinergic neurons that may contribute to these effects are illustrated in cartoon form in Figure 14. The actions of the various pharmacological agents on the different preparations used in this study are summarized in Table 1.

**Table 1:** **Summary of the effects of drugs altering cholinergic function in the various preparations used in this study**.

**Pharmacological agents**	**Neonatal rat spinal cord preparation**	**Decerebrate cat preparation**	**Acute spinal cat**	**Early chronic spinal cat**	**Late chronic spinal cat**	**Late chronic spinal rat**
Edrophonium (EDRO)	↑↑			↔		
AChE inhibitor						
Neostigmine (NEO)	↑↑					
AChE inhibitor						
EDRO + ACh				↔		
AChE inhibitor + acetylocholine						
EDRO + tubocurarine	↔					
AChE inhibitor + nicotinic antagonist						
Clonidine			↑↑			
NA agonist						
EDRO + clonidine				↑↑		
AChE inhibitor+ NA agonist						
Clonidine (low dose)			↔			
NA agonist						
Atropine		↔	↔	↑↑		
muscarinic antagonist						
Atropine + clonidine (low dose)			↑↑			
muscarinic antagonist + NA agonist						
Clonidine (low dose) + atropine			↑↑			
NA agonist + muscarinic antagonist						
Carbachol				↓↓		↓↓
cholinergic agonist						
Carbachol + atropine				↑↑		↑↑
cholinergic agonist + muscarinic antagonist						
Atropine + carbachol				↓↓		
muscarinic antagonist + cholinergic agonist						
EDRO + atropine	↑↓↓					
AChE inhibitor + muscarinic antagonist						
EDRO + telenzepine	↔					
AChE inhibitor + M_1_ antagonist						
EDRO + telenzepine (high dose)	↓↓					
AChE inhibitor + M_1_ antagonist						
EDRO + methoctramine (METHOC)	↑↓					
AChE inhibitor + M_2_ antagonist						
EDRO + METHOC (high dose)	↓↓					
AChE inhibitor + M_2_ antagonist						
EDRO + 4-diphenylacetoxy-N-methylpiperidine metiodide (4-DAMP)	↓↓				↑↑	
AChE inhibitor + M_3_ antagonist						
MT-3	↔					
M_4_ antagonist						
Mecamylamine		↔				
nicotinic antagonist						
Mecamylamine + atropine		↔				
nicotinic antagonist + muscarinic antagonist						
Dihydro-β-erythrpidine					↑↑	
nicotinic antagonist						

↑↑ , induced or enhanced locomotion;

↓↓ , blocked locomotion;

↑ , increased frequency;

↓ , decreased amplitude;

↔ *, no effect*.

### Well-coordinated locomotion is induced *in vitro* by facilitation of the endogenous cholinergic propriospinal system

The predominance of well-coordinated locomotion induced by EDRO documented in these experiments provides the first clear demonstration that facilitation of the effects of ACh released from the endogenous cholinergic propriospinal system is sufficient to activate the locomotor CPG in the isolated neonatal rat preparation. Previous experiments with application of exogenous ACh led to the conclusion that ACh-induced rhythmic activity was seldom locomotor-like, due to the predominance of ipsilateral flexor-extensor co-activation observed (Cowley and Schmidt, 1994). Other studies also suggested that cholinergic activation does not induce locomotor-like activity in the neonatal rat (Atsuta et al., 1991) or the mudpuppy (Fok and Stein, 2002) spinal cord. In the “motor functionally mature” mouse spinal cord, application of muscarine evoked irregular bursting in the ventral roots, and no locomotion (Jiang et al., 1999). According to Anglister et al. (2008), periods of well-coordinated locomotor activity can be revealed in the neonatal rat in the presence of EDRO and ACh, but only when coherent power analysis is used to analyze the variable activity produced by the network. Our detailed analysis of EDRO-induced locomotion presented here, however, clearly establishes that endogenous cholinergic activation is sufficient for the full expression of fictive locomotion. Similar results with another inhibitor of acetylcholine esterase (NEO) confirm this conclusion. Sacral application of EDRO has been shown to elicit locomotor activity in the lumbar cord (Finkel et al., 2014), and this component of a sacrocaudal afferent system capable of eliciting locomotor activity may have contributed to the results reported here. Our results suggest that using cholinesterase inhibitors allows the expression of cholinergic-induced rhythms triggered by release from the intrinsic cholinergic neurons, rather than by application of an exogenous agonist, to give rise to coordinated locomotion. This suggests that certain actions of exogenous cholinergic agonists may produce conflicting effects that actually interfere with the production of coordinated locomotion including flexor-extensor antagonist activity and right-left alternation. This could result for instance from conflicting effects of agonists on afferent inputs and central drive mechanisms. Locomotion induced by other means, such as MLR stimulation (this study) or exogenous neurochemicals such as 5-HT, dopamine and NMDA, is not antagonized by cholinergic blockers (Fok and Stein, 2002; Bertrand and Cazalets, 2011) indicating some specificity of the cholinergic antagonists used.

There is evidence for strong cholinergic influences on commissural cells (Carlin et al., 2006), and for cholinergic commissural cells that are active during locomotion (Huang et al., 2000). Some commissural cells with excitatory actions on contralateral motoneurons are cholinergic (Bertrand and Cazalets, 2011), and sacral cholinergic neurons modulate locomotor activity recorded from the lumbar segments *in vitro* (Finkel et al., 2014). A portion of these cholinergic neurons are commissural. Exogenous cholinergic agonists may have the effect of activating commissural cholinergic influences that predominate, provided the ipsilateral coordinating interneurons controlling flexor/extensor alternation are not so strongly activated.

The cholinergic input to spinal neurons includes C-terminals on motoneurons, which are now known to originate from V0c neurons that are characterized by Pitx2 expression (reviewed in Witts et al., 2014). They are active during locomotion, and they increase motoneuron firing by reducing the AHP amplitude *via* M_2_ muscarinic receptors (Miles et al., 2007; Zagoraiou et al., 2009). Pitx2 V0c neurons provide cholinergic terminals on Ia inhibitory interneurons (IaINs) (Siembab et al., 2010), the neurons involved in reciprocal inhibition from primary afferent fibers (Jankowska and Roberts, 1972). It is clear, based on the size of the terminals, that those on IaINs are not C-terminals (Siembab et al., 2010).

### M_2_ receptors control motoneuron excitability and locomotor frequency

The reduction of ENG amplitude observed in the cases of antagonists with high affinities for M_2_ receptors (atropine, methoctramine, and to a lesser extent telenzepine, and 4-DAMP) is consistent with the presence of M_2_ receptors at cholinergic terminals on motoneurons and with the finding that muscarinic receptor activation increases motoneuron excitability. The effects of various antagonists on the control of locomotion derived from the present results are summarized in Table 1. In adult mammalian motoneurons, cholinergic agents produce plateau-like responses (Zieglgansberger and Reiter, 1974), consistent with the muscarinic effects in the turtle spinal cord slice (Svirskis and Hounsgaard, 1998; Guertin and Hounsgaard, 1999). In other studies, cholinergic agonists have been shown to depolarize rat motoneurons (Evans, 1978; Jiang and Dun, 1986) and in mouse motoneurons, muscarinic receptor activation leads to an increase in excitability, manifested by an increase in f-I slope and a reduction in the post-spike afterhyperpolarization (Miles et al., 2007). This effect is due to M_2_ receptor activation at C-terminals on motoneurons (Evans, 1978; Witts et al., 2014). Other neurons involved in controlling the CPG for locomotion, currently unidentified, are implicated by the increase in locomotor frequency produced by these drugs, consistent with some tonic cholinergic suppression of CPG activity. Importantly, M_2_ receptors, but not M_1_ or M_3_ receptors, reduce glutamate release from primary afferents (Chen et al., 2014).

### M_3_ receptors are involved in cholinergic activation of the spinal locomotor CPG

The ability of the M_3_ receptor antagonist 4-DAMP (Table 1) to initially reduce the frequency of locomotion induced by EDRO and then to block it entirely at doses as low as 3 nM suggests that the cells of the locomotor network that are activated by the endogenous spinal action of ACh possess M_3_ receptors. The distribution of M_3_ receptors in the spinal cord has not been extensively described, although cells with M_3_ receptors have been observed in laminae VII, VIII and X (Wilson et al., 2004). Whether any of the cholinergic propriospinal neurons are among those with M_3_ receptors is not known. There is evidence, however, that cholinergic cells in the dorsal horn, lamina X, and the lateral intermediate zone (laminae VI) are densely labeled with M_2_ receptors (Stewart and Maxwell, 2003). The distribution of neurons in the ventral horn with M_2_ receptors has not been examined in detail, except in the case of motoneurons (Wilson et al., 2004).

In summary, the *in vitro* data presented here establish that well-coordinated locomotion can be produced in the isolated neonatal rat spinal cord, and suggest that an endogenous propriospinal cholinergic system could be a potential contributor to the recovery of locomotion after SCI due to its ability to activate the CPG for locomotion. The cholinergic propriospinal system and glutamatergic propriospinal cells endogenous to the spinal cord are among the remaining groups that might be responsible for the ability of most animal species to regain locomotor function after complete SCI (Jordan and Schmidt, 2002; Rossignol et al., 2002; Rossignol, 2006).

### Does the cholinergic propriospinal system contribute to normal locomotion or to recovery of locomotion after SCI?

Our experiments using MLR-evoked locomotion in adult cats with i.v. application of both nicotinic and muscarinic antagonists (summarized in Table 1) suggest that there is very little contribution to normal locomotion by cholinergic systems, either at the spinal or the brainstem level. This finding is not consistent with the report by Garcia-Rill et al. (1987) who showed that muscarinic receptor blockade by injecting antagonists directly into the caudal brainstem could abolish MLR-evoked locomotion. Our results show that even in cases where the stimulus site is optimum for activation of the cholinergic cells in the vicinity of the MLR (i.e., pedunculopontine nucleus: cf. Rye et al., 1987), no decrement is observed in the ability of MLR stimulation to evoke locomotion. It would appear, therefore, that the frequently observed overlap of cholinergic cells with the lowest threshold stimulation sites within the MLR (Garcia-Rill et al., 1987; Garcia-Rill and Skinner, 1988) is coincidental. Destruction of cholinergic cells in the dorsolateral tegmentum-cholinergic cell area of cats (in the region corresponding to the MLR) does not abolish the locomotor capacity of the animal once recovery from the surgery is complete (Webster and Jones, 1988). We conclude that the cholinergic contribution to the induction of locomotion is not obligatory in mammals.

The absence of effects at the spinal level is consistent with the failure of cholinergic antagonists to alter motoneuron activity in cases where the activity is not produced by cholinergic activation. The possibility that these results differ from those with intrathecal drug application because of a reduced ability of atropine and/or mecamylamine to enter the spinal cord in sufficient concentrations to alter locomotion was discussed in the Methods section of this paper, where we pointed out that the doses of these drugs chosen were equivalent to those used previously to effectively alter CNS effects. Clearly these drugs cross the blood-brain barrier in sufficient quantities to produce effects on CNS processes.

In experiments on spinal cats we found that ACh, or EDRO or carbachol, could not initiate locomotion soon after spinalization (Table 1) but rather induced an increased tonic excitability so that upon exteroceptive stimulation sustained contractions could be evoked but no locomotion. This may have been due to an excitatory effect on motoneurons. Our experiments with i.t. applications of carbachol in chronic spinal rats confirmed that carbachol dramatically interferes with spinal stepping, and this effect is reversed by atropine. To our surprise, atropine could improve the spontaneous emerging locomotor pattern in the second post-spinal week in spinal cats. We also found a synergy between clonidine and atropine at this stage after SCI, so that a small dose of clonidine which normally does not trigger locomotion could now evoke a clear locomotor pattern. Dihydro-β-erythroidine, a nicotinic antagonist, was also capable of facilitating locomotion in chronic spinal cat, suggesting both muscarinic and nicotinic control of the cholinergic propriospinal system.

This work suggests that in early days after spinalization, there is a hyper-cholinergic state of the cord that interferes with the early production of rhythmic activity. It indeed appears that adding ACh stimulation just further disrupts the ability to walk. This is consistent with observations using high dose intrathecal cholinergic agonist applications in intact rats (Yaksh et al., 1985; Gillberg et al., 1990). The increased excitability might be attributable to a direct action of cholinergic agonists on motoneurons, which can be excited by ACh acting at M_2_ receptors (Witts et al., 2014). This is consistent with our finding *in vitro* that motoneuron activity during fictive locomotion is reduced by activation of cholinergic M_2_ receptors. In addition to powerful suppression of cutaneous afferent input, possibly via M_2_ receptors (Chen et al., 2014), the cholinergic system appears to reorganize after SCI so that there is an increased cholinergic suppression of CPG activation. This would explain the absence of a locomotor facilitation by atropine early after SCI while facilitating afferent input, and the efficacy of clonidine to elicit locomotion at this stage. We interpret the fact that clonidine and atropine have a synergistic effect at very low doses at this stage as evidence that some means of activating the CPG is required at this stage, but a few weeks after SCI development of cholinergic control of some portion of the CPG allows atropine to elicit locomotor activity without the need for another means to facilitate CPG activity.

An important finding from these studies is the contrast between the locomotor promoting action of cholinergic activation in the neonatal preparation and the reversal of this effect to one that suppresses locomotion in the adult. There is no obvious explanation for this, except to speculate that there may be features of the cholinergic control that are present in the adult but absence in the neonate, and vice versa. These might include differential effects of descending pathways on the cholinergic propriospinal system, or the later development of tonic control of afferent input. There is evidence that the cholinergic terminals of the dorsal horn do not appear until sometime in the post-natal period (Phelps et al., 1984), so their influence would not be exerted in neonatal preparations.

Control of afferent input in adult animals is a mechanism revealed by our results with injections of atropine. ACh exerts both muscarinic and nicotinic control over sensory inputs (Gillberg et al., 1990; Zhuo and Gebhart, 1991; Li et al., 2002; Zhang et al., 2007; Chen et al., 2010, 2014; Hochman et al., 2010) and it is possible that early on after spinalization a powerful inhibition is exerted on afferent pathways that can be reversed by cholinergic antagonists. It is known that after spinalization, cats and rats depend much more on sensory inputs, having lost all descending inputs, particularly relying on cutaneous afferents from the foot (Bouyer and Rossignol, 2003b; Sławińska et al., 2012). Our results showing atropine effects on the recovery of locomotion and atropine enhancement of cutaneous input from the SP nerve suggest that such a mechanism underlies these results. This is also consistent with the differences between *in vitro* locomotion, which is without cutaneous input from load receptors on the foot pad, and the effects of the cholinergic agonists and antagonists during treadmill stepping in spinal cats. Such cutaneous input is an absolute requirement for locomotion in spinal cats (Bouyer and Rossignol, 2003b).

The possible role of ACh in the control of afferents that influence locomotion has not been investigated, although there is evidence for cholinergic presynaptic inhibition (Ribeiro-Da-Silva and Cuello, 1990; Hochman et al., 2010), including control of non-nociceptive cutaneous afferents. Consistent with this, M_2_ receptors are found on primary afferent fibers, and they are eliminated with dorsal rhizotomy (Li et al., 2002). It is also clear that there is postsynaptic control of sensory transmission by cholinergic neurons of the dorsal horn. For example, there is evidence for nicotinic modulation of GABAergic control of sensory input (Genzen and McGehee, 2005). The relative contributions of nicotinic and muscarinic receptors to the control of the sensory input necessary for enhancement of locomotion by cholinergic antagonists in our study is unknown, but it is clear that both types of receptors are involved in the control of sensory input to the spinal cord.

### Is the cholinergic propriospinal system modified after SCI?

Chartlon et al. (1981) and Faden et al. (1986) found that muscarinic cholinergic receptor binding is reduced after spinal cord transection. A dramatic decrease in cholinergic boutons on tail motoneurons was observed after sacral SCI (Kitzman, 2006). Skup et al. (2012) showed VAChT + boutons on TA MNs increased slightly after SCI and locomotor training, while VAChT boutons decreased dramatically on Sol MNs after spinalization, and this was partly restored with training. Kapitza et al. (2012) observed a progressive decrease in cholinergic input onto motoneuron soma, and shrinkage of cholinergic interneuron cell bodies located around the central canal after SCI. They suggested that “… reduced cholinergic input on motoneurons is assumed to result in the rapid exhaustion of the central drive required for the performance of locomotor movements in animals and humans….” These observations predict that the effect of SCI should be a reduced excitability of motoneurons and other targets of the cholinergic propriospinal cells. Our results directly contradict these predictions, and show evidence for a hyper-cholinergic state after SCI. They suggest the use of cholinergic antagonists as a potential treatment of recovery of function after SCI. They further suggest that the suppression of afferent input to the spinal cord by ACh *via* both muscarinic and nicotinic receptors is the key to understanding the role of ACh in motor control after SCI.

## Conclusions

Our results show a potent cholinergic control of locomotor activity emerges after spinal cord injury, but in contrast to expectations, it was neither a decrease in motoneuron excitability, as predicted by recent findings, nor a recruitment of cholinergic propriospinal cells to account for stepping in spinal animals. Instead, a hyper-cholinergic state that supressed locomotor activity emerged, and this could be reduced with cholinergic antagonists to facilitate locomotion. Figure 14 summarizes the cholinergic neurons that might account for our results. V0c (Pitx2) and commissural cholinergic neurons (CIN) contact motoneurons directly and increase their excitability, while unknown cholinergic interneurons, likely from the partition cell and central canal cluster cell groups, project to CPG neurons and account for the cholinergic locomotor rhythm control (LRC). Dorsal horn cholinergic interneurons known to inhibit cutaneous afferent input and produce analgesia, either by presynaptic inhibition or by controlling other interneurons (not shown) that are responsible for afferent input control (AIC) are also illustrated. The AIC cholinergic cells are the most likely ones to account for the ability of cholinergic antagonists to facilitate locomotion. We believe such a population of AIC cholinergic neurons must tonically inhibit afferent feedback necessary for locomotor activity in spinal animals. It is likely that controlling the activity of these neurons will provide a new opportunity for restoring locomotion after injury.

## Author contributions

Serge Rossignol and Larry M. Jordan conceived and designed the experiments on neonatal rats and spinal cats. B. R. Noga designed and carried out the experiments on decerebrate cats with systemic drug applications. J. R. McVagh carried out the *in vitro* experiments on neonatal rat spinal cord. J. Provencher, H. Leblond, Larry M. Jordan, and Serge Rossignol performed the experiments on chronic spinal cats, while A. M. Cabaj, H. Majczyński, Urszula Sławińska, and Larry M. Jordan carried out the intrathecal drug application experiments on chronic spinal rats. Larry M. Jordan, Serge Rossignol, Urszula Sławińska, J. R. McVagh, H. Leblond and B. R. Noga wrote various aspects of the paper. All the authors discussed the results and accepted the final version of this manuscript.

### Conflict of interest statement

The authors declare that the research was conducted in the absence of any commercial or financial relationships that could be construed as a potential conflict of interest.

## Abbreviations

4-DAMP: 4-diphenylacetoxy-N-methylpiperidine methiodide
5-HT: 5-hydroxytryptamine, serotonin
ACh: acetylcholine
AChE: acetylcholine esterase
AChR: acetylcholine receptor
aCSF: artificial cerebral spinal fluid
AF: adductor femoris muscle
AHP: afterhyperpolarization
AIC: afferent input control
BB: biceps brachii muscle
b.w.: body weight
CIN: commissural interneurons
CNF: nucleus cuneiformis
CNS: central nervous system
CPG: central pattern generator
EDRO: edrophonium
EMG: electromyogram
ENG: electroneurogram
GL: gastrocnemius lateralis muscle
GM: gastrocnemius medialis muscle
IaINs: Ia inhibitory interneurons
I.D.: internal diameter
IP: iliopsoas muscle
i.p.: intraperitoneal
i.t.: intrathecal
L_2_, L_3_, L_4_, L_5_: lumbar spinal cord segments
LRC: locomotor rhythm control
M_1_, M_2_, M_3_, M_4_: muscarinic receptors
METHOC: methoctramine
MLR: Mesencephalic Locomotor Region
MNs: motoneurons
MT-3: muscarinic toxin-3
NEO: neostigmine
NMDA: *N*-Methyl-D-aspartate
O.D.: outside diameter
PPN: pedunculopontine nucleus
PT: posterior tibial nerve
Pitx2: transcription factor
s.c.: subcutaneously
SCI: spinal cord injury
SD: standard deviation
Sol: soleus muscle
SP: superficial peroneal nerve
Srt: sartorius muscle
St: semitendinosus muscle
T9, T10, T13: thoracic spinal cord segments
TA: tibialis anterior muscle
TB: triceps brachii muscle
VAChT: vesicular acetylcholine transporter
VL: vastus lateralis muscle
WAG rats: Wistar Albino Glaxo rats.
